# Neonatal apneic phenotype in a murine congenital central hypoventilation syndrome model is induced through non‐cell autonomous developmental mechanisms

**DOI:** 10.1111/bpa.12877

**Published:** 2020-08-04

**Authors:** Diego Alzate‐Correa, Jillian Mei‐ling Liu, Mikayla Jones, Talita M. Silva, Michele Joana Alves, Elizabeth Burke, Jessica Zuñiga, Behiye Kaya, Giuliana Zaza, Mehmet Tahir Aslan, Jessica Blackburn, Marina Y. Shimada, Silvio A. Fernandes‐Junior, Lisa A. Baer, Kristin I. Stanford, Amber Kempton, Sakima Smith, Caroline C. Szujewski, Abby Silbaugh, Jean‐Charles Viemari, Ana C. Takakura, Alfredo J. Garcia, Thiago S. Moreira, Catherine M. Czeisler, José J. Otero

**Affiliations:** ^1^ Division of Neuropathology Department of Pathology The Ohio State University College of Medicine Columbus OH USA; ^2^ Department of Physiology and Biophysics Institute of Biomedical Science University of São Paulo São Paulo Brazil; ^3^ Department of Pharmacology Institute of Biomedical Science University of São Paulo São Paulo Brazil; ^4^ Department of Physiology and Cell Biology The Ohio State University College of Medicine Columbus OH USA; ^5^ Department of Internal Medicine The Ohio State University College of Medicine Columbus OH USA; ^6^ Institute for Integrative Physiology Grossman Institute for Neuroscience Quantitative Biology and Human Behavior The Committee on Neurobiology The University of Chicago Chicago IL USA; ^7^ P3M Team Institut de Neurosciences de la Timone UMR 7289 AMU‐CNRS Marseille France

**Keywords:** apnea, chemosensation, congenital central hypoventilation syndrome (CCHS), *Nkx2.2*, *PHOX2B*, respiratory rhythm‐generating networks

## Abstract

Congenital central hypoventilation syndrome (CCHS) represents a rare genetic disorder usually caused by mutations in the homeodomain transcription factor *PHOX2B*. Some CCHS patients suffer mainly from deficiencies in CO_2_ and/or O_2_ respiratory chemoreflex, whereas other patients present with full apnea shortly after birth. Our goal was to identify the neuropathological mechanisms of apneic presentations in CCHS. In the developing murine neuroepithelium, *Phox2b* is expressed in three discrete progenitor domains across the dorsal‐ventral axis, with different domains responsible for producing unique autonomic or visceral motor neurons. Restricting the expression of mutant *Phox2b* to the ventral visceral motor neuron domain induces marked newborn apnea together with a significant loss of visceral motor neurons, RTN ablation, and *preBötzinger* complex dysfunction. This finding suggests that the observed apnea develops through non‐cell autonomous developmental mechanisms. Mutant *Phox2b* expression in dorsal rhombencephalic neurons did not generate significant respiratory dysfunction, but did result in subtle metabolic thermoregulatory deficiencies. We confirm the expression of a novel murine *Phox2b* splice variant which shares exons 1 and 2 with the more widely studied *Phox2b* splice variant, but which differs in exon 3 where most CCHS mutations occur. We also show that mutant *Phox2b* expression in the visceral motor neuron progenitor domain increases cell proliferation at the expense of visceral motor neuron development. We propose that visceral motor neurons may function as organizers of brainstem respiratory neuron development, and that disruptions in their development result in secondary/non‐cell autonomous maldevelopment of key brainstem respiratory neurons.

## Introduction

Although ventilation is a necessary biological function beginning at birth and ending at death, the pathophysiological mechanisms by which Central Nervous System (CNS) ventilation control dysfunction develops in newborns continues to elude neuropathologists. Significant insights into CNS ventilatory control have come from studying human diseases such as Congenital Central Hypoventilation Syndrome (CCHS). CCHS (OMIM #209880) represents a rare human genetic disorder of the Autonomic Nervous System (ANS) caused by heterozygous mutations in *PHOX2B* (1, 66). CCHS patients show monotonous breathing rhythms that do not change in response to altered levels of CO_2_ or O_2_, especially during non‐rapid eye movement (NREM) sleep (29, 67). CCHS patients present with a spectrum of CNS respiratory control pathophysiologies, ranging from deficiency in chemosensation during unassisted breathing, to the most severe manifestations of overt apnea in the neonatal setting that requires life‐sustaining mechanical ventilation regardless of vigilance states. Neonatal apnea may present in CCHS patients regardless of *PHOX2B* mutation type, and the developmental mechanisms by which neonatal apneic phenotypes manifest in some CCHS patients but not in others is not well understood.

Breathing maintains homeostatic pH and arterial partial pressure of carbon dioxide (pCO_2_) and partial pressure of oxygen (pO_2_). This is achieved by a complex interaction of multiple rhombencephalon‐derived neural circuits. Central chemoreceptors, which are capable of monitoring and sensing blood pH and CO_2_ levels as well as modulating the respiratory rhythm, stimulate respiration in response to hypercapnia (22). Excitatory neurons of the retrotrapezoid nucleus (RTN), derived embryonically from the dorsal dB2 rhombencephalic progenitor domain, mediate central chemoreception (51). Murine RTN neurons, which are situated ventral to the Facial Motor Nucleus (nVII) on the lateral medullary surface, express *Vglut2, Phox2b*, galanin and neuromedin B, among other markers (55). During acidification (which parallels an increase in arterial pCO2), RTN neurons send signals to the ventral respiratory column to regulate ventilation. Neuropathological findings in rodent CCHS models demonstrating RTN dysgenesis support the notion that the deficient chemoreflex of CCHS patients may result from defects in RTN function (13). However, RTN neuronal loss does not itself induce full apneic phenotypes in mice (41) which suggests that the neuropathological mechanisms by which patients develop neonatal apnea distinct from the mechanisms which induce defective chemosensation.

Data from human patients and murine CCHS models lead to an obvious question. Namely, does CCHS pathophysiology result from dysfunctional Phox2b‐targetted gene expression in known Phox2b‐positive respiratory centers (such as the RTN), or does CCHS disease result from developmental pathologies in non‐respiratory progenitor cells? Given that *Phox2b* expression occurs across a wide swath of cells during embryogenesis, we proceeded to express a highly penetrant NPARM *Phox2b* mutation in two progenitor lineages that do not give rise to the RTN. We found that targeting mutant *Phox2b* into the visceral motor neuron progenitor domain, achieved by breeding *Nkx2.2^Cre^ mice to Phox2b^∆8^* mice, resulted in a significant population of pups showing apnea shortly after birth with electrophysiological abnormalities in the PreBotzinger complex, as well as deficiencies in a spectrum of rhombomeric nuclei not developmentally derived from *Nkx2.2*‐expressing progenitors. We provide evidence for a non‐cell autonomous mechanism to CCHS neuropathology.

## Materials and Methods

### Experimental animals and animal husbandry procedures

Procedures utilizing mice were performed in accordance with The Ohio State University Institutional Animal Care and Use Committee guidelines (protocol number: 2012A00000162‐R1) and in accordance with the University of Chicago Institutional Animal Care and Use Committee guidelines (protocol number: 72486). Information regarding the strains of mice used including strain number and genotyping details are delineated in Supporting Table S1. In this developmental screen, *Phox2b^+/∆8^‐*expressing mice were compared to their *Phox2b^+/+^* littermates. *Nkx2.2^Cre^* (kind gift of Dr. Michel Matis, Rutgers University (63)) and *Olig3^Cre^* (kind gift of Dr. Yasushi Nakagawa, University of Minnesota (62)) mice were bred with a *ROSA^TdTomato^* transgenic reporter line (Ai14) (36) to determine which structures are developmentally dependent on *Nkx2.2* or *Olig3* expression, respectively. *Cre*‐inducible inhibitory Designer Receptors Exclusively Activated by Designer Drugs (DREADD) (*ROSA^+/hM4Di^)* (68) mice were bred with *Nkx2.2^Cre^* and *Vglut2^Cre^* (61) drivers in order to selectively silence with clozapine‐N‐oxide (CNO) (# C0832, Sigma‐Aldrich) treatment neurons expressing the inhibitory, *hM4Di DREADD* receptor. An intersectional genetic strategy in which the Intersectional RC::FLTG mouse line (46) was bred with the *Nkx2.2^Cre^* and Phox2b‐FLPo (26) Fsuppmouse lines in order to evaluate *Nkx2.2* and *Phox2b* embryonic derivation. Genotyping was verified by PCR (Terra PCR Direct Red Dye Premix #639286, Clontech Laboratories) using primers detailed in Table S1.

### Histology, immunohistochemistry and microscopy

#### Histology and immunostaining

Mice were anesthetized using a mixture of ketamine (100 mg/kg) and xylazine (10 mg/kg) in sterile saline and perfused transcardially, first with 10 mL of PBS, then with 5 mL of 4% paraformaldehyde (PFA) in PBS. Pup brains were drop‐fixed in 4% PFA in DEPC‐PBS at 4ºC overnight, equilibrated in 30% sucrose/DEPC‐PBS at 4ºC until tissue sank to bottom of container and OCT embedded. OCT‐embedded pup brains were cryosectioned at thicknesses optimized for each antibody. For immunostaining, sections exceeding 25 μm were processed as floating sections in a 96‐well plate. Brain tissue was antigen retrieved with 10mM citrate buffer (pH 6.0) in an 80° C water bath for 30 minutes then washed with PBS + 0.1% Triton X‐100 (#02300221, MP Biomedicals) three times for 10 minutes. Tissue was then blocked with 10% horse serum (#06750, Stem Cell Technologies) and 0.1% Triton X‐100 in PBS before incubation with primary antibody solution (5% horse serum, 0.1% Triton X‐100 in PBS) overnight at 4°C. Tissue was washed with PBS + 0.1% Triton X‐100 three times for ten minutes before incubation with secondary antibody solution (5% horse serum, 0.1% Triton X‐100) and counterstained with DAPI 1:1000 (#D1306, Invitrogen) for two hours at room temperature in the dark. Sections were first washed with PBS two times then with dH_2_O once for 10 minutes and coverslips were mounted with ProLong Gold Antifade Mountant (#P36934, Invitrogen). Our standard operating procedures for embryo analyses were similar, but with some modifications delineated in the Supporting Information.

Analyses of the nucleus of the solitary tract (NTS) in *Olig3^Cre^, Phox2b^Δ8^* and control littermate P0 pups were challenging due to different atlases using varied terminology. The terms commissural NTS, lateral NTS, and medial NTS are utilized as shown in the Allen Developing Brain Atlas. The term rostral NTS, which is not present in the Allen Brain Atlas, is denoted to represent a population of NTS cells described by Paul Gray that are *LMX1B*‐derived (21). Using the Allen Developing Brain Atlas for orientation, sister sections of the hindbrain, cryosectioned at 40 μm, were stained for PHOX2B and Choline Acetyltransferase (ChAT). Dorsal hindbrain structures analyzed, using Neurolucida’s cell counter program, were PHOX2B^+^, ChAT^‐^ including the NTS and the area postrema (AP). All antibody details are delineated in Table S2.

#### Microscopy and image analysis

Fluorescent images were captured on a Carl Zeiss Axio Imager Z.1 with LSM700 confocal microscope with a motorized stage using 10x/0.3 NA, 20X, and 40x/1.3 NA Oil DIC objectives. For cell cycle analyses and visualization of hindbrain pathology in *Phox2b^Δ8^‐*expressing mice, single optical slices or Z‐stacks were taken at 20X or 40x magnification. Tiled images were tiled with 10% overlap and stitched by Zen software (2011, Black, v. 7,0,0,285) into a 3D image. Cell quantitation was performed using different techniques optimized for each experiments as follows: (i) for the *Olig3^cre^* experiments were carried out in Neurolucida software (v 11.11.2, MicroBrightField) using the cell detector capable of automatic cells counts within a user‐specified region, (ii) for quantitation of P0/1 Nkx2.2cre, Phox2bD8 neuropathology and BrdU quantitation, we the manual cell counter system from FIJI, (iii) for quantitation of E12.5 emrbyos and Phox2b nuclei segmentation of E10.5 embryos, we implemented custom R scripts through “imager” or “EBImage” packages. Details are delineated in the Supporting Information. For RNA quantitation of *Phox2b‐202* FISH, the entire thickness of analyzed cells was included in analysis by capturing 25 Z sections with a step size of 0.3µm. Images were converted into a single Z‐plane by maximum intensity projection in FIJI (v1.0, RRID: SCR_002285) and *Phox2b‐202* puncta manually counted in FIJI using the cell counter plugin.

### Cell cycle analysis

For cell cycle length analyses, an E10.5 pregnant dam carrying *Nkx2.2^Cre^, Phox2b^Δ8^* embryos were injected intraperitoneally (i.p.) with 5‐Chloro‐2′‐deoxyuridine thymidine (CldU) at 50 mg/kg (#C6891, Sigma‐Aldrich). The dam was then injected i.p. with 5‐Ethynyl‐2'‐deoxyuridine (EdU) at 50 mg/kg (#E10187, Thermo Fisher Scientific) 2 hours after CldU administration. Thirty minutes after EdU injection, E10.5 embryos were collected via cesarean section. Cryosections were taken at 14 µm, unmasked via antigen retrieval with sodium citrate 10mM, and stained with pertinent antibodies. Note that CldU was immunolabeled with anti‐BrdU antibody (Novus Biologicals, #NB500‐169), and therefore all figure legends are referred to as BrdU represent CldU signals. EdU labeling was achieved using an Alexa Fluor^™^ 488 Click‐iT Kit (#C10337, Thermo Fisher Scientific) as specified in the manufacturer’s protocol. Control mice were littermates of *Nkx2.2^Cre^, Phox2b^Δ8^* mice that do not express mutant *Phox2b^Δ8^* (ie, they are *Phox2b^+/+^*). Detailed workflows of the image analysis workflows are available in the Supporting Information.

### Gene expression analysis

*RT‐qPCR:* Quantitative reverse transcription polymerase chain reaction (RT‐qPCR) was carried out using the SuperScript^™^ III First‐Strand Synthesis (#18080051, Thermo Fisher Scientific) and SYBR^™^ Green PCR Master Mix (#4309155, Thermo Fisher Scientific) for a two‐step RT‐qPCR assay on a Step One Plus RT‐PCR system (# 4376600, Applied Biosystems). Step One v2.3 software (Applied Biosystems) was used for comparative Ct analysis, with mouse Gapdh as the endogenous control. Primers for RT‐qPCR are delineated in the Supporting Information.

#### RNA fluorescence in situ hybridization

To selectively target the mRNA of either *Phox2b* splice variant 1 (*Phox2b‐201*) or the putative *Phox2b* alternative splice variant 2 (*Phox2b‐202*), we utilized the Stellaris Probe Designer (http://www.biosearchtech.com/stellaris‐designer) to create two custom sets of fluorescently labeled oligonucleotides. The first set, *Phox2b‐201*, consisted of 34 oligos mapped to the 3’UTR and C‐terminal end of *Phox2b* exon 3 splice variant 1 and labeled with CAL Fluor 610. These oligos were designed against the mouse *Phox2b* exon 3, and because *Phox2b^Δ8^* mutant mice express human *PHOX2B* exon 3 instead of mouse *Phox2b* exon 3, *Phox2b‐201* oligos do not hybridize to *Phox2b^Δ8^* transcripts. The second set, Phox2b‐202, was comprised of 21 oligos mapped to the 3’UTR and C‐terminal end of *Phox2b* exon 2 splice variant 2 and labeled with Quasar 670. For quality control, ShipReady Stellaris FISH probes against *Gapdh* fluorescently labeled with Quasar 570 dye (#SMF‐3002‐1, LGC Biosciences) were used. Sequences of oligos included in each custom probe sets are listed in Table S3. RNA FISH hybridizations were performed overnight according to the manufacturer’s protocol for frozen mouse brain tissue.

### Physiological testing

#### Newborn respiration analysis in mutant *Phox2b^Δ8^* mice

Respiration was analyzed in neonatal pups from both *Nkx2.2^Cre^, Phox2b^Δ8^ and Olig3^Cre^, Phox2b^Δ8^ genotypes* using a mouse pup plethysmography chamber (#987365, DSI) and QT preamplifier (#894002, DSI). P0 pups were placed in the chamber in the head‐out configuration and acclimated for 1 minute before respiration was recorded for 10 minutes. Breathing in adult mice was monitored using a whole‐body plethysmographer (#992541, DSI) with the Buxco Finepointe 2‐site controller (#993582, DSI). Finepoint software was used (v2.4.7.10175) to record respiration parameters including, but not limited to, respiratory frequency (f_R_, breaths per minute), tidal volume (V_T_, mL/kg) and minute ventilation (V_E_, mL/min/kg). Quantitation of the number and duration of apneic occurrences was done manually by a blind observer. Apnea was defined as the absence of plethysmographic flow signal for more than two expected respiratory cycles (15, 27). Justification for pooling control genotypes is presented in the Supporting Information.

#### Chemogenetic experiments (newborn pups)

hM4Di expressing P1 pups underwent their first recording at baseline for 10 minutes. Following the initial recording, pups underwent CNO administration by intraperitoneal injection (i.p.) followed by 60 minutes at room air to equilibrate. Pups were then placed in the plethysmographer and respiration recorded for another 10 minutes. Control mice were littermates of *ROSA^+/hM4Di^* mice that do not express Cre‐recombinase.

#### Chemogenetic experiments (adult mice): respiration analysis following neuronal circuit inhibition in *ROSA^+/hM4Di^* adult mice

hM4Di expressing adult mice underwent acclimatization to the plethysmographer for 15 minutes/day for 3 days. Plethysmograph recordings lasting 10 minutes prior to CNO administration via intraperitoneal (i.p.) injection (0.25 mg/kg) represented our baseline values. After 60 minutes, respiration was recorded and analyzed during a 10‐minute recording interval. Data processing and analysis are detailed in the Supporting Information.

#### Electrophysiological slice recordings

Brainstem slices containing the preBötC were prepared from *Nkx2.2^Cre^, Phox2b^Δ8^* and control mice (postnatal Day 0) as described previously (18). Briefly, the dorsal face of the isolated brainstem was glued to an agar block and submerged in artificial cerebrospinal fluid (aCSF, ~4°C) equilibrated with Carbogen (95% O2, 5% CO2). Transverse slices were made through the brainstem until anatomical landmarks, such as the inferior olive and hypoglosssal nucleus (XIIn), were identified. A single slice (600 ± 30 μm) containing the preBötC and XIIn was taken.

The composition of aCSF was as follows (in mM): 118 NaCl, 3.0 KCl, 25 NaHCO_3_, 1 NaH_2_PO_4_, 1.0 MgCl_2_, 1.5 CaCl_2_ and 30 d‐glucose. When equilibrated with Carbogen at ambient pressure, the aCSF had a pH of 7.40‐7.45 and an osmolarity of 308 ± 4 mOSM. Before recording, the extracellular KCl concentration was raised to 8.0 mM to induce rhythmic activity from the preBötC. Extracellular population activity was recorded with a glass suction pipette (tip resistance < 1 MΩ) filled with aCSF that was positioned over the ventral respiratory column containing the preBötC. Recorded signals were amplified 10 000X, filtered (low pass, 1.5 kHz; high pass, 250 Hz), rectified, and integrated using an electronic filter. Recordings were acquired in pCLAMP software (Molecular Devices, Sunnyvale, CA) and were analyzed *post hoc* in Clampfit software (version 10.2). Calculation of Irregularity score for a given metric was performed *post hoc* and previously described by (17).

### Statistical modeling and data analysis

Descriptive and inferential statistics were performed in Microsoft Excel or R Studio. Student’s *t*‐test for pairwise comparison or ANOVA with Tukey’s HSD correction for multiple comparisons was used as appropriate for each experiment as indicated in figure legends. Final figures were prepared using Adobe Illustrator © 2019 v24. Detailed data analytical pipelines are presented in the Supporting Information.

## Results

### Selective expression of NPARM *Phox2b* in *Nkx2.2*‐derived cells results in an apneic presentation at birth

**Figure 1:**
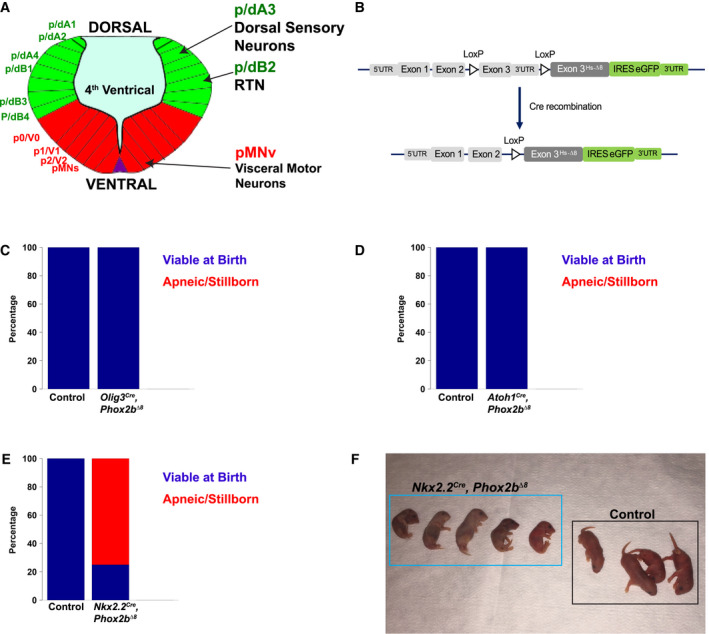
*Experimental design and mouse developmental screen*. **A**. Artistic rendering of rhombomere 4 of an E10.5 mouse embryo. The developing rhombencephalon can be subdivided into dorsal progenitor domains (green) and ventral domains (red). Within each domain, specific neuronal and glial populations are generated. *Phox2b* is expressed in 3 progenitor domains depicted on the right: dA3, dB2, and pMNv. These progenitor domains give rise to dorsal sensory neurons, retrotrapezoid nucleus RTN, and visceral motor neurons, respectively. **B**. Transgenic mouse strategy. The humanized NPARM *Phox2b* mouse mutation is located in exon 3. *Cre*‐mediated recombination replace the endogenous murine exon 3 with a human exon3 harboring an eight‐nucleotide deletion (Hs‐Δ8). **C**‐**E**. Developmental screen of mice to identify apneic/stillborn presentations. Stacked bar graphs show proportion of pups viable at birth in blue, with apneic/stillborn pups in red. Genotype is denoted on the bottom of each bar graph. This screen (**C**) derives from 28 control pups and 44 mutant pups spread over 10 litters, (**D**) derives from 10 control pups and 18 mutant pups spread over six litters, (**E**) derives from 10 control pups and 12 mutant pups. (F) Photograph of a representative *Nkx2.2^Cre^, Phox2b^Δ8^* litter showing absent or limited respiratory movements and cyanosis at birth.

**Figure 2:**
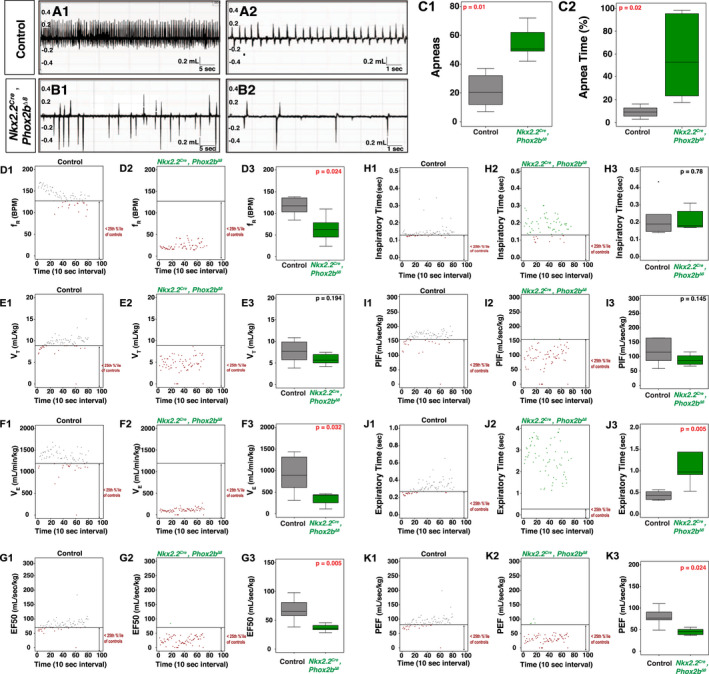
*Respiratory Physiology in newborn* Nkx2.2^cre^, Phox2b^Δ8^ *pups*. **A** and **B**. Wave forms obtained from head out plethysmography configuration. Genotype designated on left. **A1** and **B1** are 1‐minute‐long traces demonstrating the marked differences in respiratory frequency between control and *Nkx2.2^Cre^, Phox2b^Δ8^* mice. **A2** and **B2** are 16‐second‐long breathing traces demonstrated the waveforms of each breath for control and *Nkx2.2^Cre^*, Phox2b*^Δ8^* mice. **C1** and **C2** show the number and duration of apneas, respectively. Analysis of respiratory patterns is shown as follows: **D1**‐**D3** show frequency (f_R_), **E1**‐**E3** show tidal volume (V_T_), **F1**‐**F3** show minute ventilation (V_E_), **G1**‐**G3** show EF50, **H1**‐**H3** show inspiratory time (Ti), **I1**‐**I3** show peak inspiratory flow (PIF), **J1**‐**J3** show Expiratory time (Te), and **K1**‐**K3** show peak expiratory flow. In each series, an example animal is plotted for each genotype. Each plot shows a line that represents the 25th percentile of pooled control data, with each data point lying out of the 25th percentile colored in red (note the significant number of red data points in the mutants). Box‐whisker plots show the median as the bar, the box is the interquartile range, and the whiskers are 1.5 times the interquartile range. Control is in gray, *Nkx2.2^Cre^, Phox2b^Δ8^* is green. Corrected *P*‐values are shown in the top right of each boxplot, with red indicating tests with statistical significance. *Nkx2.2^Cre^, Phox2b^Δ8^* show significantly decreased frequency, minute ventilation, EF50, peak expiratory time and an increased expiratory time. n = 6 control, n = 6 *Nkx2.2^cre^*, *Phox2b^Δ8^* mice obtained from two distinct litters for these analyses.

We utilized a spectrum of *Cre* drivers that develop into key breathing structures (see schematic in Figure 1A,B: *Atoh1^Cre^*, *Olig3^Cre^* and *Nkx2.2^Cre^* induce recombination in cells giving rise to the CO_2_‐sensitive neurons of the RTN (50, 51, 64), dorsal rhombencephalic sensory neurons (58), and rhombencephalic ventral visceral motor neuron progenitor domain (pMNv) (7, 69), respectively. The results of these crossings are shown in Figure 1C‐E, which demonstrates that *Phox2b^Δ8^* expression in *Nkx2.2*‐derived cells results in neonatal apneic presentations in mice (Figure 1F) (*P* = 0.0005 by Fisher’s Exact Test). As pathological and functional evaluation of mutant *Phox2b* expression in *Atoh1^Cre^*‐expressing cells (37) has been extensively evaluated in the scientific literature (12, 13, 14, 24, 48), we, therefore, focused our investigations on expressing *Phox2b^Δ8^* in progenitor populations derived from the ventral (*Nkx2.2*‐derived) and dorsal (*Olig3*‐derived) poles as their respective contributions to autonomic nervous system development and function have been less explored. For breathing pattern, we subjected newborn *Nkx2.2^cre^*, *Phox2b^Δ8^* pups to restrained (head out) plethysmography (Figure 2). An example of the respiratory pattern in control and mutant animals is shown at two different timescales in Figure 2A,B; note that the *Nkx2.2^cre^, Phox2b^Δ8^* animals show irregular patters with frequent and longer apneas (Figure 2C). Frequency (f_R_) and minute ventilation (V_E_) were significantly diminished in *Nkx2.2^cre^*, *Phox2b^Δ8^* animals (Figure 2D,F). We also noted significant neurophysiological findings during expiration, including increased expiratory time (Te) (Figure 2J), with decreased EF50 (Figure 2G) and peak expiratory flow (PEF) (Figure 2K). However, tidal volume (V_T_) (Figure 2E), mean inspiratory time (Ti) (Figure 2H) and peak inspiratory time (PIF) (Figure 2I) were not different between groups. In view of these findings, we performed linear regression analysis of these data by placing the frequency (f_R_) as the response variable with both expiratory time (Te) and inspiratory time (Ti) as the predictor variables. The linear regression data are reported in Table S4. We note that the *Nkx2.2^Cre^, Phox2b^Δ8^* animals showed a significantly repressed linear relationship between f_R_ and Te. Specifically, the model predicts that each unit increase of Te reduces f_R_ by 109 breaths per min (BPM) in the controls, whereas in the *Nkx2.2^cre^*, Phox2b^Δ8^ animals each unit increase in Te reduces f_R_ by *only* 35 BPM. Of note, the Ti did not show a significant linear relationship with f_R_ in the *Nkx2.2^cre^*, *Phox2b^Δ8^* animals, indicating that in these mutants f_R_ could not be predicted by Ti, yet showed no change in *mean* Ti between mutant and control groups (Figures 2H1 and 3). We conclude that expressing *Phox2b^Δ8^* in the *Nkx2*.2 lineage induces an apneic phenotype at birth as well as an abnormal respiratory pattern.

**Figure 3:**
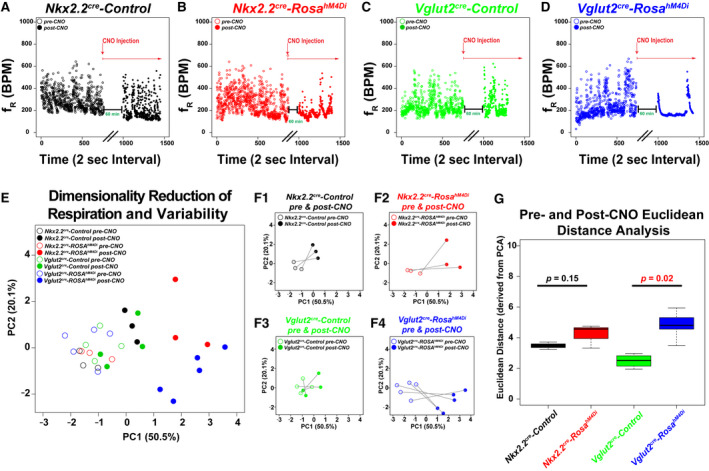
*Respiratory Physiology of* Nkx2.2^Cre^ hM4Di *inhibitory DREADD mice*. **A**‐**D**. Dot plots show genotype on top, frequency on Y‐axis (breaths per minute), and time (2 sec bins) on X‐axis for sample animals before (open circle) and after (closed circle) i.p. injection of CNO (0.25 mg/kg). In each dot plot, the experimental paradigm is shown delineating the CNO treatment. Note that data were appended from pre and post‐CNO recordings and plotted continuously on the same graph for ease of representation (black parallel lines on X‐axis denote breaking of the continuous scale, with the post‐CNO recording arbitrarily beginning at x = 1000). *Nkx2.2^Cre^*, *ROSA^+/h4MDI^* animals are plotted in red for dot plots, PCA, and bar graphs, *Nkx2.2^Cre^*, Controls are plotted in black, *Vglut2^Cre^*, are plotted in green, and *Vglut2^Cre^*, *ROSA^+/h4MDI^* animals are plotted in blue. **E**. Principal component analysis of all of the ventilation data and respiration variability data derived from Poincaré analysis are graphed along PC1 (50.5% of variance) and PC2 (20.1% of variance). Color coded legend is in the top right for **E**‐**F**. **F**. For ease of representation, each group is graphed in isolation before and after CNO treatment along PC1 and PC2. F1 = Nkx2.2cre Control, F2 = Nkx2.2cre, ROSAh4MDi, F3 = Vglut2cre control, F4 = Vglut2cre, Rosah4MDi. **G**. The Euclidean distance along all of the principal components was calculated for each animal before and after CNO treatment, and then graphed as a box‐whisker plot based on genotype. Box plots show solid black line to represent the median, the box represents in the interquartile range, and the whiskers represent 1.5 times the interquartile range. *P*‐values of no significance are shown in the box plots in black font, with corrected *P*‐values of < 0.05 shown in red. For *Nkx2.2* experiments, *Nkx2.2^Cre^, ROSA^+/h4MDI^* n = 3, control animals n = 3. For *Vglut2* experiments, *Vglut2^Cre^, ROSA^+/h4MDI^* n = 4, control n = 5.

Unlike the *Nkx2.2^cre^, Phox2b^Δ8^* animals, which show perinatal lethality, the viability of *Olig3^cre^, Phox2b^Δ8^* animals permitted us to test the autonomic responses to a wide array of physiological challenges across the lifespan. These data are illustrated in Figures S2 and S3. *Olig3^cre^, Phox2b^Δ8^* animals do not show changes in respiratory parameters during the postnatal period relative to controls. P21 and adult animals showed no significant difference from controls during hypercapnia and hypoxic challenges (Figure S2), nor were changes noted during hyperoxic hypercapnia (Figure S3). We conclude that *Phox2b^Δ8^* expression in *Olig3*‐derived cells does not alter the hypercapnic nor hypoxic respiratory chemoreflex.

### Chemogenetic silencing of *Nkx2.2‐*derived neurons does not induce apnea

Having established that *Phox2b^Δ8^* expression in progenitors derived from *Nkx2.2*‐ (but not *Olig3*‐derived) leads to neonatal respiratory dysfunction, we next asked if modulating *Nkx2.2*‐derived cells through chemogenetic‐mediated silencing would result in apneic phenotypes similar to *Phox2b^Δ8^* expression. We utilized recombination with the *Vglut2^Cre^* transgenic mouse as an expected positive control since *VGLUT2* shows RTN expression (2, 39) and is expressed in ~45% of *DBX‐1*‐derived preBötC neurons (3, 16). To achieve this, we interbred *Nkx2.2^Cre^* or *Vglut2^Cre^* mice with *ROSA^+/hM4DI^*, a *Cre*‐inducible inhibitory DREADD mouse line (*ROSA^+/hM4Di^*). In *ROSA^+/hM4Di^* mice, neurons derived from *Cre*‐positive cells express the hM4Di DREADD receptor. Cells harboring the hM4Di DREADD receptor become hyperpolarized after treatment with the ligand clozapine‐N‐oxide (CNO). For both the *Nkx2.2^Cre^* and *Vglut2^Cre^* mouse experiments, we performed baseline plethysmography measurements of littermate control *ROSA^+/hM4Di^* mice before and 1‐hour post‐i.p. CNO administration, and compared the results to *Cre*‐positive, *ROSA^+/hM4Di^* mice. Results from our assays are illustrated in Figures 3 and S4‐S6 (Online Resource 1). Note that in *Vglut2^Cre^* experiments performed on adult mice, application of CNO to *Cre*‐positive *ROSA^+/hM4Di^* mice significantly decreased the variability of breaths per minute (Figure 3A‐D), which we attribute to CNO‐dependent changes in CNS ventilation control. These experiments resulted in a high dimenstionality dataset. We were successful in reducing dimensions of our data by implementing principal component analysis on the respiratory and respiratory variability data. Specifically, our initial dataset of 48 data dimension per animal were reduced to 30 principal components (PC, see details in Supporting Information). Figure 3E‐F shows coordinates along PC1 and PC2 pre‐CNO (open circles) and post‐CNO (closed circles). This analysis permitted us to calculate the Euclidean distance for each animal pre‐CNO and post‐CNO across the 30 PC dimensions, a measure representing the total CNO effect on each animal relative to the total variance noted in the dataset amongst all genotypes. As can be seen in Figure 3G, although *Nkx2.2^cre^*, *ROSA^+/hM4Di^* animals showed a trend to increased Euclidean distance, it did not meet our threshold of significance. In contrast the significantly different Euclidean distance was noted in the *Vglut2^cre^*, *ROSA^+/hM4Di^* animals. We also evaluated each parameter head to head using traditional approaches (Figures S4‐S6). The following parameters showed no change in mean between pre‐CNO and post‐CNO administration in the *Cre*‐positive *ROSA^+/hM4Di^* mice versus control mice in both the *Nkx2.2^Cre^* and *Vglut2^Cre^* experiments: frequency (f_R_, BPM), tidal volume (V_T_, mL/kg), minute ventilation (V_E_, mL/min/kg), inspiratory time (Ti, s), expiratory time (Te, s), peak inspiratory flow (PIF, mL/s/kg), peak expiratory flow (PEF, mL/s/kg), expiratory flow at 50% expired volume (EF50, mL/s/kg), end expiratory pause (EEP, ms), and end inspiratory pause (EIP, ms). In summary, chemogenetic silencing of glutamatergic neurons decreases variances of respiratory pattern. We further conclude that silencing *Nkx2.2‐*derived cells does not phenocopy the *Nkx2.2^Cre^, Phox2b^Δ8^* phenotype.

*Nkx2.2* encodes a transcription factor that regulates development of visceral motor neurons, serotonergic neurons, and glia (44). It has been proposed by some authors that serotonergic neurons play a role in rhythmogenesis early in development, which gradually shifts to a chemosensory role in adult mice (6). To test potential effects of serotonergic cell fate, we generated *Nkx2.2^Cre^*, *ROSA^+/hM4Di^* newborn animals and silenced *Nkx2.2^Cre^* derived neurons with CNO and tested respiratory function by head out/restrained plethysmography (Figure S7). Similar to the adult data, we found no change in frequency, tidal volume, minute ventilation, Ti, nor EF50 in *Nkx2.2^Cre^, ROSA^+/hM4Di^* newborn pups. We conclude that chemogenetic silencing *Nkx2.2*‐derived circuits does not decrease respiratory frequency in adults nor in newborn pups.

### *Nkx2.2^Cre^, Phox2b^Δ8^* mice show diffuse brainstem neuropathology and weakened inspiratory drive from the preBötC, whereas *Olig3^cre^, Phox2b^Δ8^* mice show normal dorsal Phox2b‐neuron specification

**Figure 4:**
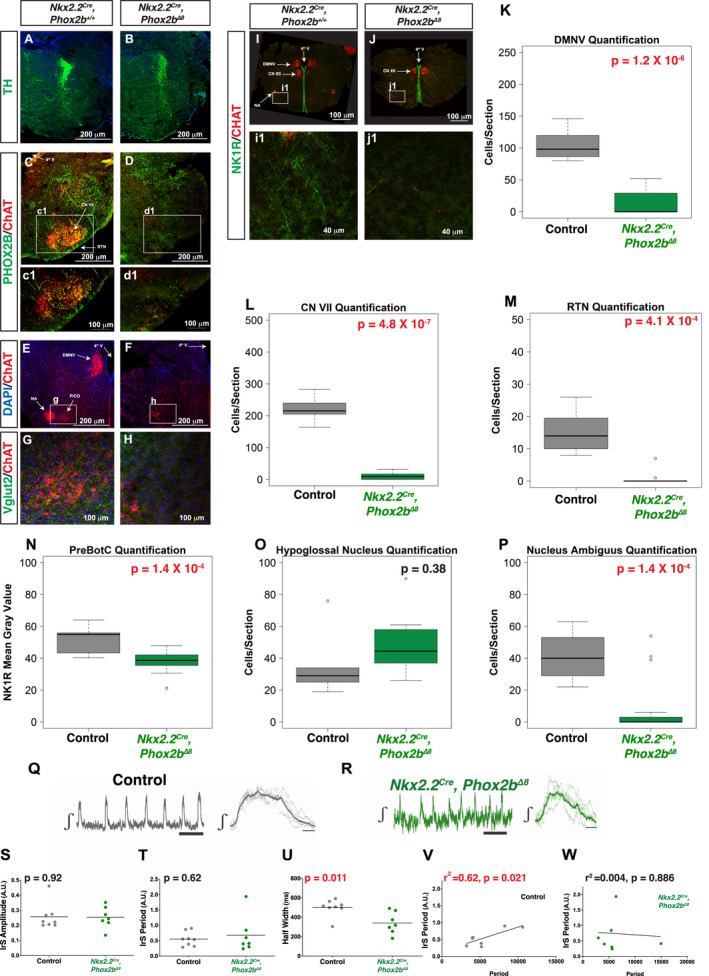
*Brainstem Neuropathologies of* Nkx2.2^Cre^; Phox2b^Δ8^ *mice are accompanied by weakened inspiratory drive from the preBötC*. **A** and **B**. No apparent defect in the Locus coeruleus (TH+) in *Nkx2.2^Cre^, Phox2b^Δ8^* mice compared to controls noted. **C** and **D**. VII nucleus (ChAT in red) and RTN with PHOX2B‐positive neurons (green) at the ventral medullary surface. **E**‐**H**. Significant dysgenesis of dorsal motor nucleus of vagus nerve and nucleus ambiguus at the level of the post‐inspiratory complex (PiCo) respiratory oscillator (PICO is delineated in higher magnification in panels g and h for control and mutant mice, respectively). Panels **I**‐**J** Dorsal motor nucleus of the vagus nerve, nucleus ambiguus and a decreased intensity of NK1R‐immunoreactive cells noted. **K**‐**P**. Box plots show solid black line to represent the median, the box represents in the interquartile range, and the whiskers represent 1.5 times the interquartile range. *P*‐values of no significance are shown in the box plots in black font, with corrected *P*‐values of < 0.05 shown in red. In box plots the parameter tested is labeled on Y‐axis corresponding to either the number of ChAT‐positive cells/section (**K**, **L**, **O**, **P**), PHOX2B positive cells/section (**M**), or NK1R Mean gray Value (**N**). Genotypes are delineated on X‐axis. TH = Tyrosine Hydroxylase, ChAT = Choline Acetyltransferase, NK1R = Neurokinin 1 Receptor, VGLUT2 = vesicular glutamate transporter‐2. *Nkx2.2^Cre^, Phox2b^Δ8^* n = 10 images obtained from three pups, control n = 5 images obtained from two pups. **Q**‐**W**. Representative image of the integrated inspiratory rhythm from isolated preBötC *left*, and the mean inspiratory burst (thick gray trace) of seven individual inspiratory bursts (thin gray traces) *right*, from a control preBötC isolated in a brainstem slice (**Q**); representative image of the integrated inspiratory rhythm from isolated preBötC *left*, and the mean inspiratory burst (thick green trace) of seven individual inspiratory bursts (thin green traces) *right* from a preBötC isolated in a brainstem slice from a *Nkx2.2^Cre^, Phox2b^Δ8^* animal (**R**). While no differences were observed when comparing the irregularity score (IrS) of amplitude (**S**, *P* = 0.92) and period (**T**, *P* = 0.62) between the preBötC from control (n = 8) and *Nkx2.2^Cre^, Phox2b^Δ8^* (n = 7) animals, halfwidth of preBötC burst from *Nkx2.2^Cre^, Phox2b^Δ8^* animals has a significant shorter duration (**U**, *P* = 0.011). No difference in the mean IrS of period observed. Variance in IrS period was different between the two groups (*F* = 6.538, *P*‐value for F test to compare variance = 0.026). Positive relationship existed between IrS of period and period in the control group (*r* ^2^ = 0.62, P = 0.021) (V). In the *Nkx2.2^Cre^, Phox2b^Δ8^* group, the relationship was flat (*r* ^2^ = 0.004, *P* = 0.886) (**W**).

In order to fully understand *Nkx2.2^Cre^*, *Phox2b^Δ8^* neuropathology, we generated timed pregnant females and evaluated E18.5 mouse brains. We focused our evaluation on the morphology of well‐documented respiratory centers or *Phox2b*‐derived nuclei, including locus coeruleus (LC), dorsal motor nucleus of the vagus nerve (DMNV), cranial nerve VII nucleus (nVII), retrotrapezoid nucleus (RTN), nucleus of the solitary tract (NTS), nucleus ambiguus (NA) and PreBötC. These structures are identified by a combination of immunohistochemical markers within a neuroanatomical context and are illustrated in Figure 4. We note that in contrast to *Hprt^Cre^*, *Phox2b^Δ8^* mice which show loss of LC (Nobuta et al., 2015), *Nkx2.2^Cre^*, *Phox2b^Δ8^* mice generate a well‐formed LC (Figure 4A,B). The following brainstem nuclei were anatomically unaffected: the NTS, the median raphe (MR), and the PHOX2B‐positive cells located in the parvicellular reticular nucleus and vestibular nucleus (data not shown). In contrast, we found a near ablation of the following structures: CN VII (Figure 4C,D), RTN (Figure 4C,D), DMNV (Figure 4I‐J) and the NA (Figure 4E,F). We also noted a decreased NK1R mean gray value in the PreBötC region (Figure 4I1‐J1,N). We also noted a decrease in Choline Acetyltransferase (ChAT)‐positive cells in the area of the post‐inspiratory complex (PiCo, Figure 4E‐H). To quantitate the differences observed, we counted the number of ChAT‐positive cells of the DMNV (Figure 4K), CN VII (Figure 4L, Hypoglossal Nucleus (Figure 4O) and NA (Figure 4P). In addition, we quantified the number of PHOX2B positive cells in the RTN (Figure 4M). These data indicate the *Phox2b^Δ8^*expression in *Nkx2.2*‐derived cells leads to widespread cytoarchitectural changes in key respiratory control centers.

To address the possibility that the *Phox2b^Δ8^* mutation destabilizes inspiratory drive, we conducted extracellular recordings from the preBötC isolated in brain slices prepared from control (Figure 4Q) and *Nkx2.2^cre^*, *Phox2b^Δ8^* (Figure 4R) animals. While the regularity of amplitude (Figure 4S) and period (Figure 4T) was similar between groups, the burst duration was smaller in inspiratory rhythms from *Nkx2.2^Cre^, Phox2b^Δ8^* animals (Figure 4U). Examining the relationship between irregularity of rhythmogenesis and period reveals that period irregularity correlates with cycle period (Figure 4V) yet this association was not present in the *Nkx2.2^Cre^, Phox2b^Δ8^* group (Figure 4W). Thus, while the mutant preBötC was capable of generating inspiratory rhythms, the weaker inspiratory drive from the preBötC of *Nkx2.2^Cre^, Phox2b^Δ8^* animals produces irregular activity independent of the frequency of rhythmogenesis that is normally found in control animals. These data demonstrate preBötzinger complex dysfunction in the *Nkx2.2^cre^*, *Phox2b^Δ8^* mice.

**Figure 5:**
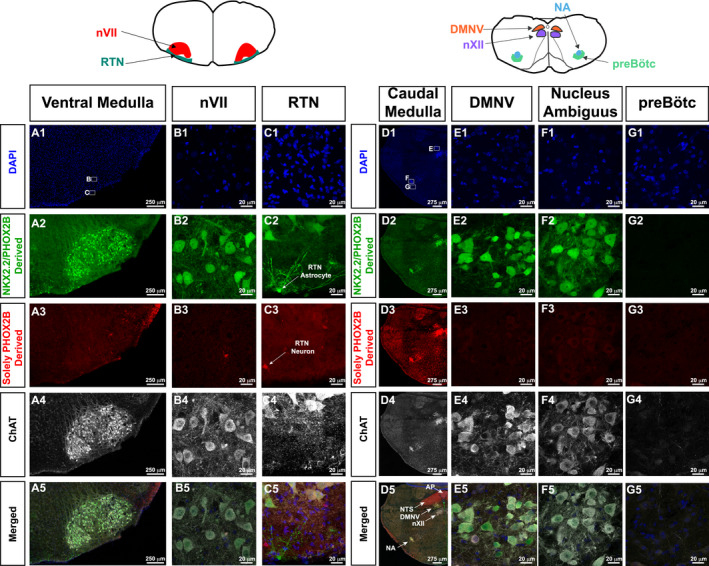
*Lineage tracing of* Nkx2.2/Phox2b‐*derived and* Phox2b‐*derived cells*. For orientation, cartoons of brainstem anatomy are illustrated atop of the panels, with left cartoon pertaining to **A**‐**C**, whereas right cartoon pertains to d‐g. In this intersectional approach, GFP expressing cells show derivation from both *Nkx2.2* and *Phox2b* expressing cells (second row from top), and tdTomato expressing cells are solely derived from PHOX2B expressing progenitor cells (third row from top). ChAT (white in fourth and fifth row from top) highlights cholinergic neurons. In **A1** and **D1**, small white boxes delineate where the high magnification photomicrographs in the respective panels were obtained. In Panel **C2**, white arrow indicates a *Nkx2.2*‐derived, *Phox2b*‐derived astrocyte. Panel **C3**, white arrow indicates *Phox2b‐*derived RTN neuron. Panel **D5** shows the anatomy of key medullary structures including area postrema (AP, *Phox2b*‐derived, ChAT‐negative), nucleus of the solitary tract (NTS, *Phox2b*‐derived, ChAT‐negative), dorsal motor nucleus of the vagus (DMNV, Nkx2.2/*Phox2b*‐derived, ChAT‐positive), hypoglossal nucleus (nXII, not derived from *Phox2b* nor *Nkx2.2*, ChAT‐positive), and nucleus ambiguus (NA, *Nkx2.2*/*Phox2b*‐derived, ChAT‐positive). RTN neurons are not derived from *Nkx2.2*, and preBötC neurons, which do not express tdTomato nor GFP, do not show *Nkx2.2* nor *Phox2b* derivation.

We next performed lineage tracing studies to identify the *Phox2b*, and *Nkx2.2*‐derived lineages so that we could evaluate the neuropathology within an embryonic cell lineage context. To achieve this, we utilized an intersectional genetic strategy. By this strategy, *flp*‐recombinase expression alone induces *tdTomato* expression, but if *flp*‐recombinase and *Cre*‐recombinase are expressed within the same developmental lineage, the tdTomato cassette is excised, permitting *GFP* expression. We performed analyses on P7 *Phox2b‐FLP0, Nkx2.2^Cre^*, *RC::FLTG* animals. Note that the cholinergic cells of the nVII cranial nerve showed GFP expression in this animal, indicating that nVII neurons are derived from progenitor cells that express *Nkx2.2* and *Phox2b* (Figure 5A,B). However, in the RTN, small neurons at the ventral surface show tdTomato expression (Figure 5C3 arrow), whereas a subset of RTN astrocytes show dual derivation of *Nkx2.2* and *Phox2b* (10). In the caudal medulla, the dorsal motor nucleus of the vagus nerve (DMNV) and nucleus ambiguus (Figure 5E‐F), a well‐characterized *Phox2b*‐expressing neuron cluster (33), shows derivation from both *Phox2b* and *Nkx2.2*‐expressing progenitor cells. The area postrema and NTS (Figure 5D5, arrows) show derivation from only *Phox2b*, whereas the preBötC neurons show no evidence of derivation from *Nkx2.2* nor *Phox2b*‐derived progenitor cells (Figure 5G1‐G5). We, therefore, conclude that the loss of visceral motor neurons in *Nkx2.2^Cre^*, *Phox2b^Δ8^* animals (ie, nVII, nucleus ambiguus, and dorsal motor nucleus of vagus nerve) results from cell autonomous expression of mutant *Phox2b^Δ8^*. However, the respiratory centers analyzed (RTN, and preBötC) do not show *NKX2.2* derivation. We propose that the agenesis/dysfunction of these structures is due to non‐cell autonomous effects on the development of these structures.

We next tested if we could identify any neuropathological findings in the *Olig3^Cre^, Phox2b^Δ8^* mice in brainstem structures. To this end, we procured postnatal Day 0/1 (P0/1) pup brains and performed neuroanatomical analyses. Choline Acetyltransferase (ChAT) immunofluorescence was used for orientation throughout our brainstem sections. These findings are illustrated in Figure S8. We note that the dorsal rhombencephalic *Phox2b*‐derived structures, including NTS, DMNV and area postrema (AP) were not detrimentally affected. Unsurprisingly, the nVII and RTN were unaffected as well. However, we noticed an increase of dorsal *PHOX2B+* cells in the rostral brainstem of *Olig3^Cre^*, *Phox2b^Δ8^* mice relative to controls (compare Figure S8A1,A2, quantified in Figure S8I). In view of these findings, we then performed lineage tracing by interbreeding *Olig3^Cre^* mice with *ROSA^tdTomato^* mice. *Olig3* demarcates the dorsal progenitor domain in the early neural tube (60), developing spinal cord (40) and rhombencephalon (58). *Olig3^−/−^* mice show loss of NTS (35, 58) yet preservation of cerebellar architecture. No recombination was noted in the RTN, the nucleus ambiguus, and in preBötC neurons. Our experiments demonstrated robust recombination in cerebellum (Figure S8D‐E), the parabrachial nucleus, and a subset of NTS cells (ranging from 5 to 15% of PHOX2B‐positive NTS cells, quantified in Figure S8J). We also noted robust expression of tdTomato in the dorsal ependymal cells of the central canal as well as in the area postrema (AP) (Figure S8F1,F2,G1,G2,H1,H2), a circumventriculate organ that regulates a diverse array of physiological processes, including metabolism (reviewed by (49)). We conclude that only a subset of PHOX2B‐positive NTS neurons are derived from *Olig3* progenitor cells in our model, and that the AP shows strong *Phox2b/Olig3 d*erivation.

Given the role that the AP plays in autonomic homeostasis, we were interested in exploring other potential physiological perturbations that could occur in *Olig3^Cre^, Phox2b^Δ8^* mice. Lesions in the AP reportedly show defects in cardiovascular regulation, including attenuation of hypotensive pharmacologic agents (8), modulation of hypertension‐inducing diets in rats (5), and regulation of mean arterial pressure (56). Furthermore, AP neurons possess both nicotinic and muscarinic acetylcholine receptors (45, 57). We, therefore, performed baseline blood pressure measurements before and following treatment with the muscarinic acetylcholine receptor agonist carbachol. These results are shown in Figure S9. Mean arterial pressure, systolic blood pressure, and diastolic blood pressure were unaffected, relative to control, in *Olig3^Cre^, Phox2b^Δ8^* mice. Furthermore, *Olig3^Cre^, Phox2b^Δ8^* mice responded normally to carbachol administration, showing significant lowering in blood pressure relative to baseline. We conclude that *Phox2b^Δ8^* expression in *Olig3*‐derived cells does not affect cardiovascular response to muscarinic agonism.

The AP has a well‐documented role in metabolic regulation (reviewed by Waterson et al., (65)). We, therefore, performed metabolic analysis in a Comprehensive Lab Animal Monitoring System (CLAMS, Columbus Instruments) in *Olig3^Cre^, Phox2b^Δ8^* mice during different physiological states. These factors included fed/fasted, dark/light, mutant/control, and room temperature/4 °C. We analyzed our data by performing linear regression models where VO_2_, VCO_2_, heat energy expenditure, or Activity were response variables to the aforementioned factors. The summary of these linear regression models are shown in Tables S5‐S8. Although *Olig3^Cre^, Phox2b^Δ8^* genotype as a factor was itself not found to significantly alter the response variables tested, we did find a significant interaction of *Olig3^Cre^, Phox2b^Δ8^* status with CLAMS performed at 4º C for VO_2_, VCO_2_ and energy expenditure, but not activity. VO_2_, VCO_2_ and energy expenditure were increased in response to cold exposure in mutant, but not WT mice. Specifically, the linear regression model showed that *Olig3^Cre^, Phox2b^Δ8^* status increased VO_2_, VCO2 and energy expenditure by 14.3% (*P* = 0.005), 16.6% (*P* = 0.0036) and 19% (*P* = 0.0044), respectively. This intriguing association may be due to dysfunction of PHOX2B‐positive AP neurons in *Olig3^Cre^, Phox2b^Δ8^* mice. We propose that metabolic regulation and temperature homeostasis shows dysfunction in *Olig3^Cre^, Phox2b^Δ8^* mice.

### *Phox2b* splice variant expression in *Nkx2.2^Cre^, Phox2b^Δ8^* mice

**Figure 6:**
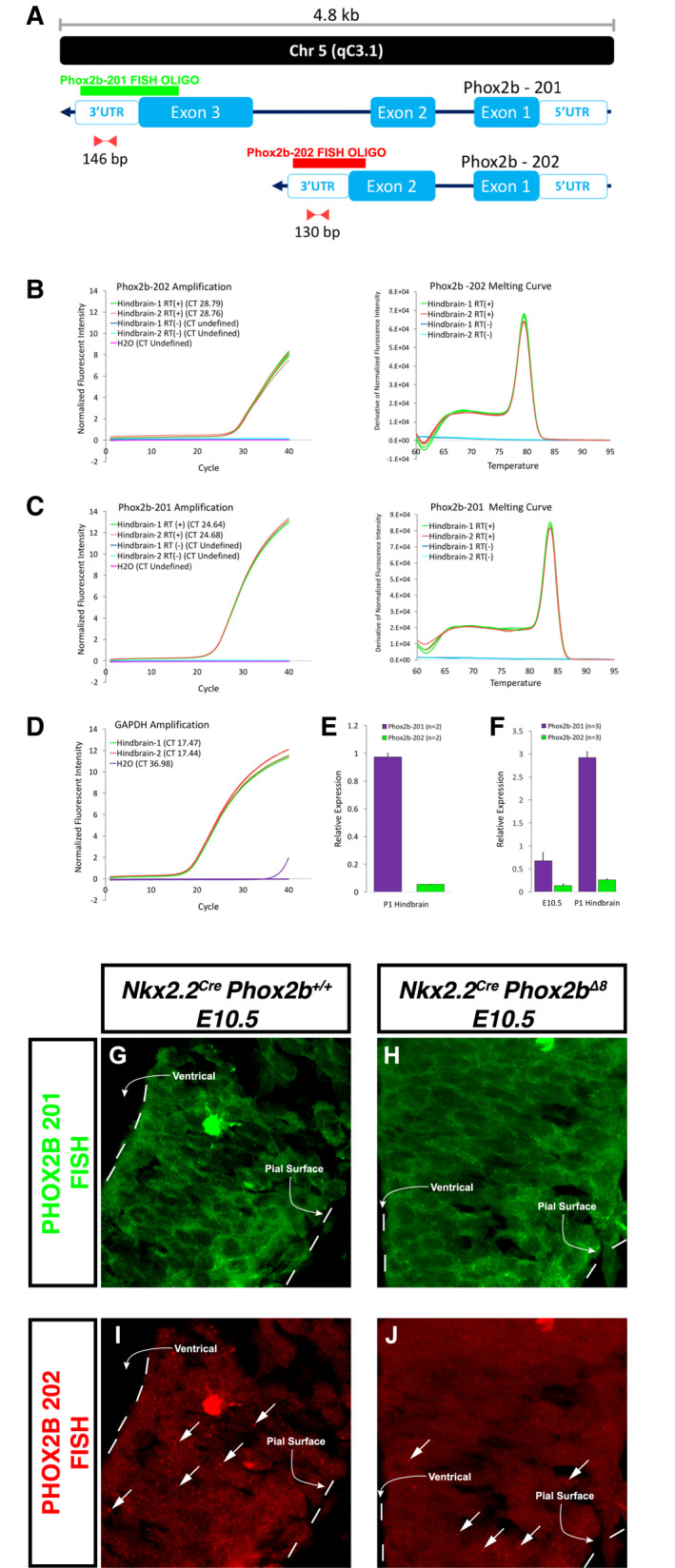
*Transcriptional analysis of PHOX2B expression in developing hindbrain*. **A**. Schematic representation of mRNA for two splice variants described for *Phox2b*. Splice variants are annotated automatically by the Ensembl database and manually curated by the HAVANA group. Also depicted are the relative binding location and the amplicon length of primer pairs and probes designed to amplify and detect unique sequences at the 3’UTR region of each splice variant. B‐C Amplification (left) and melting curves (right) obtained after a RT‐PCR assay for *Phox2b‐202* (**B**) and *Phox2b‐201* (**C**) using cDNA samples from hindbrains of two mice at P1. Amplification curves described the cycle threshold (CT) for each sample and melting curves show the presence of a single amplicon for each primer pair. RNA samples that were not subjected to Reverse Transcriptase reaction (RT(‐)) were used as negative controls. **D**. Amplification curve for Gapdh showing the respective CT for each sample. **E**. Relative expression of *Phox2b*‐202 and *Phox2b*‐201 obtained after analysis of the amplification curves for the two hindbrain samples. **F**. Expression of the two *Phox2b* splice variants is detected on E10.5 embryos and P1 mice. Probes targeting *Phox2b‐201* (G‐H) and *Phox2b‐202* (**I**‐**J**) in control and *Nkx2.2^cre^, Phox2b^Δ8^* embryos, respectively. Arrows in **I**‐**J** point to punctuated morphologies that are characteristic of *Phox2b‐202*. The bright structure in **G** and **I** represents an erythrocyte.

*In‐silico* analysis of the *Phox2b* locus demonstrated that, in addition to the well‐characterized 3‐exon containing *Ph0x2b* mRNA fragment, a smaller transcript exists lacking the complete DNA‐binding homeodomain (Ensembl number NSMUSG00000012520). Hereon we refer to the well‐characterized, full‐length transcript as *Phox2b* splice variant 1 (*Phox2b‐201*), and the shorter mRNA transcript *Phox2b* splice variant 2 (*Phox2b‐202*), as illustrated in Figure 6A. We note that many commonly utilized antibodies in the scientific literature detect an N‐terminal PHOX2B epitope in common to proteins produced from *Phox2b‐201* and potentially *Phox2b‐202*, or a C‐terminal Phox2b epitope unique to *Phox2b‐201* protein products (41, 43). No antibody capable of detecting *Phox2b*‐*202* expression has been confirmed or published to our knowledge in the scientific literature. Therefore, we generated oligonucleotide primers to confirm the *in‐silico* prediction of *Phox2b‐201* and ‐*202*. We demonstrate detection of this transcript at E10.5 and at P1 in Figure 6B‐F. Note that under control developmental circumstances, the transcript levels of *Phox2b‐201* are larger than *Phox2b‐202* at E10.5. However, by postnatal Day 1, *Phox2b* splice variant 201 is further elevated relative to splice variant 202. We conclude from these qRT‐PCR studies that the *in‐silico* prediction of *Phox2b‐202* mRNA expression is accurate, and that the ratio of Phox2b‐202/Phox2b‐201 changes dynamically during development.

Having confirmed the existence of *Phox2b‐202*, we then sought to design FISH probes that could recognize both endogenous transcripts. This was necessary because our N‐terminal PHOX2B antibody cannot distinguish between proteins produced from *Phox2b‐201*, *Phox2b‐202*, or *Phox2b^Δ8^* protein products. In our design, we took advantages of the differences between these transcripts as the oligos designed against *Phox2b‐ 201* would not bind to the mutant exon 3 in *Phox2b^Δ8^*. We tested for the expression of *Phox2b‐202* in *Nkx2.2^Cre^, Phox2b^Δ8^* mice at E10.5 by RNA FISH. *Phox2b‐201* oligonucleotide morphology was characterized by significant granular texture in the cytoplasm of neuroepithelial cells (Figure 6G‐H). In contrast, the splice variant 202 signals showed less granular cytoplasm, with markedly large punctated staining scattered throughout the neuroepithelium (Figure 6I‐J). We identified both splice variants 201 and 202 in the *Nkx2.2^Cre^, Phox2b^Δ8^* mice and littermate controls in the ventral neuroepithelium at E10.5, with no detectable difference noted between mutant and control groups at this age.

### *Phox2b^Δ8^* expression in the *Nkx2.2*‐derived progenitor domain results in embryonic patterning changes of the ventral neuroepithelium

**Figure 7:**
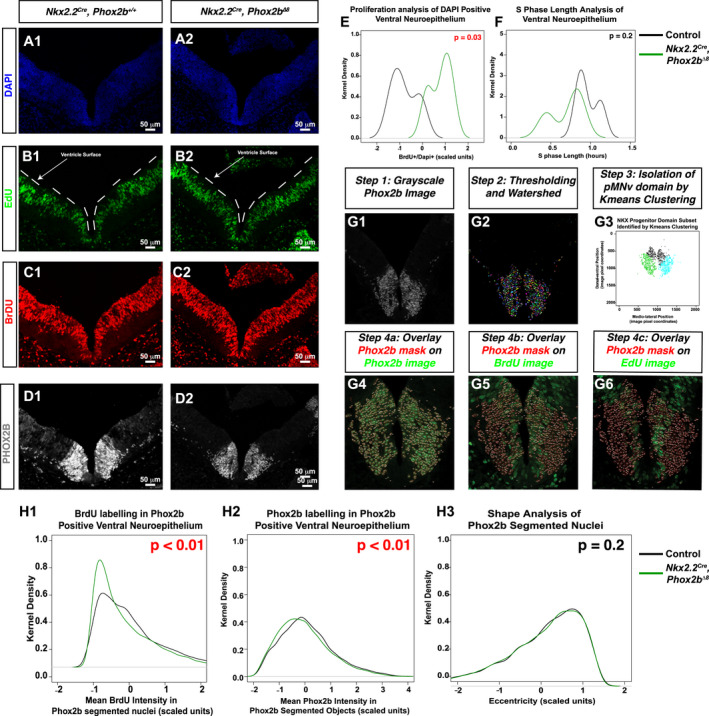
Nkx2.2^Cre^, Phox2b^Δ8^ *mice exhibit altered cell proliferation and disordered ventral neural tube differentiation*. **A**‐**D**. Sections from E10.5 embryos at the level of rhombomere 4. A CldU‐EdU pulse chase was performed in order to determine whether cell proliferation was altered in mice. CldU was administered 2 hours before administering EdU. Thirty minutes later, the animal was sacrificed and the E10.5 embryos were collected. CldU was detected using anti‐BrdU antibody, and EdU detected via click‐it chemistry. White lines in EdU image denote the ventricle surface. **E**. Proportion of BrdU positive cells over DAPI was performed, scaled, and plotted using the kernel density estimator on Y‐axis and scaled value on the X‐asix. A paired *t*‐test demonstrated that *Nkx2.2^Cre^, Phox2b^Δ8^* mice underwent significantly more cell proliferation during the 2.5‐hour incubation with CldU than controls in the pMNv domain, although S‐phase length estimation was not significantly (**F**). (**G1**‐**G6**) Image analysis workflow from Phox2b image (**G1**), to segmentation (**G2**), isolation of pMNv domain by Kmeans clustering (**G3**), and overlaying Phox2b segmented nuclei as masks over the Phox2b channel (**G4**), BrdU channel (**G5**), and EdU channel (**G6**). **H1**. Kernel density estimator functions of the distribution of BrdU intensities in Phox2b segmented objects. Mutant mice show a higher proportion of Phox2b segmented objects with low BrdU mean intensities (top right shows *t*‐test *P*‐value comparing mean BrdU intensities amongst all Phox2b segmented objects between control and experimental groups). **H2**. A slight leftword shift in distribution of Phox2b mean intensities in Phox2b segmented objects in shown (top right shows *t*‐test *P*‐value comparing mean BrdU intensities amongst all Phox2b segmented objects between control and experimental groups). **H3**. Eccentricity kernel density estimation function is superimposed on control, with no statistical significance noted.

We next tested the extent to which *Phox2b^Δ8^* expression in *Nkx2.2*‐derived cells disrupts the embryonic patterning and embryonic cell cycle analysis in *Nkx2.2^Cre^, Phox2b^Δ8^* mice. We generated timed pregnancies to obtain *Nkx2.2^Cre^, Phox2b^Δ8^* mice and control littermates at E10.5. The analysis focused on rhombomere 4 (r4). At E10.5, rhombomere 4 shows full thickness *Phox2b* expression throughout ventricular and mantle zone, with Phox2b/Islet1‐positive motor neurons having exited cell cycle occupying the mantle zone, and Phox2b/Nkx2.2‐positive progenitor cells occupying the ventricular zone (Figure 7A1‐D2).

We found that at E10.5, a trend in the reduction in the number of ISLET‐positive cells in the mantle zone of *Nkx2.2^Cre^, Phox2b^Δ8^* embryos. This trend is represented as a decrease in percent of positively expressed ISLET cells labeled with DAPI in the mutants which approaches statistical significance by E12.5 (Supporting Table S9 – Online Resource 1). These data indicate that the statistically significant loss of ISLET1 positive visceral motor neurons identified in our E18.5 experiments may begin as early as E10.5 in *Nkx2.2^Cre^, Phox2b^Δ8^* mice. Proliferation assays occurred by pulsing animals with CldU (2.5 hours) and EdU (0.5 hours), a technique permitting calculation of S‐phase time in neural progenitor cells (47) as well as S‐phase localization of progenitor cells (23). Note that the brief EdU pulse‐labeled progenitor cells distant from the ventricular surface, whereas CldU pusling delinates signals spanning both EdU and nuclei at the ventricular surface (Figure 7B1,B2,C1,C2, note that CldU is revealed using anti‐BrdU antibody). We demarcated the ventral neuroepithelium by marking the dorsal and ventral extremes of the ventral Phox2b immunoreactive region (a region that included both Phox2b+ and Phox2b− cells), and in that region counted the number of BrdU positive, EdU positive and BrdU positive/EdU negative nuclei for S‐phase cycle estimation. Note that the distribution of %BrdU positive nuclei (ie, (BrdU+/Dapi+)%) is higher relative to control, indicating that *Nkx2.2^Cre^, Phox2b^Δ8^* mice have more proliferation in the ventral neuroepithelim relative to controls without significant changes in S‐phase (Figure 7E,F).

**Figure 8:**
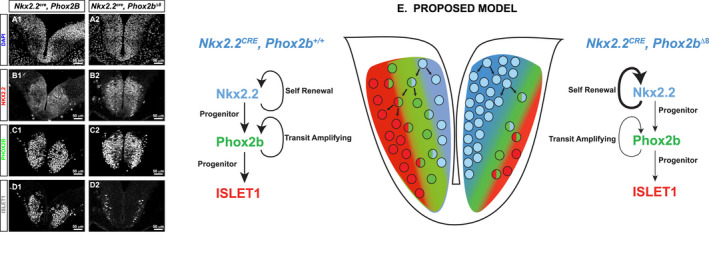
*Experimental model of visceral motor neuron loss in CCHS*. *Nkx2.2^Cre^, Phox2b^Δ8^* E10.5 embryos and controls were stained with antibodies against N‐terminal PHOX2B, NKX2.2 **and** ISLET1 (**A**‐**E**). Note the decrease in ISLET1‐positive motor neurons is noted in D1. **E**. Proposed model of visceral motor neuron maldevelopment. Wild‐type scenario is artistically rendered on left side, which illustrates a self‐renewing *Nkx2.2* progenitor stage, giving rise to a *Phox2b* positive proliferative pool with transit amplifying characteristics, which then give rise to terminally differentiated ISELT1 positive cells. In CCHS, the proliferative PHOX2B population is reduced, and there is an expansion of the *Nkx2.2* proliferative population, resulting over time to a net decreased production of ISLET1 positive cells.

We next tested proliferation within the r4 Phox2b+ region. To achieve this, we developed an image analysis workflow which objectively segments Phox2b+ nuclei (Figure 7G1‐G6, see details in Supporting Information). In brief, we used the segmented objects (representing Phox2b+ nuclei, Figure 7G1) as a mask which were overlaid over the Phox2b, EdU and BrdU image channels. This permitted us to extract imaging features in each Phox2b immunoreactive object over all the Phox2b, EdU and CldU channels. These features included intensity, Haralick texture and shape features. As an exploratory step, we generated class labels of either “experimental” (representing segmented objects from *Nkx2.2^cre^, Phox2b^Δ8^* mice) or “control” groups for each segmented object. Next, we trained random forest, adaboost, support vector machine, and LDA classification algorithms on a training set composed of 70% of randomly sampled objects across all animals. We note that decision tree‐based algorithms random forest and adaboost were able to show significant classification accuracy on the test set data (confusion matrixes shown in Figure S10A‐D). The random forest model showed BrdU mean pixel intensity as the most important features in classification of the Phox2b segmented objects as belonging to experimental versus control groups (Figure S10E). After further evaluation, we noted that the Phox2b segmented objects from *Nkx2.2^cre^, Phox2b^Δ8^* mice had a larger distribution of low BrdU pixel intensities (note the significant leftward shift in distribution of BrdU mean pixel intensity from *Nkx2.2^cre^, Phox2b^Δ8^* segmented objects, Figure 7H1) with a slightly lower, albeit reaching our threshold of significance, intensity of Phox2b (Figure 7H2). In contrast, the morphological feature Eccentricity showed no statistical difference between groups, indicating that the auto‐segmentation procedure had created similar object types during automated analysis (Figure 7H3). These data imply that the Phox2b+ progenitor population undergoes less proliferation in *Nkx2.2^cre^, Phox2b^Δ8^* mice. Furthermore, found that at E10.5, a trend in the reduction in the number of ISLET‐positive cells in the mantle zone of *Nkx2.2^Cre^, Phox2b^Δ8^* embryos (Figure 8A‐D). This trend is represented as a decrease in percent of positively expressed ISLET cells labeled with DAPI in the mutants which approaches statistical significance by E12.5 (Table S9). These data indicate that the statistically significant loss of ISLET1 positive visceral motor neurons identified in our E18.5 experiments may begin as early as E10.5 in *Nkx2.2^Cre^, Phox2b^Δ8^* mice.

## Discussion

### Proposed developmental mechanism for apneic phenotype in CCHS

The objective of this study was to elucidate developmental mechanisms by which some CCHS presentations manifest with full apnea. We tested the hypothesis that the apneic CCHS phenotype may be elicited through maldevelopment in different embryonic progenitor domains. We discovered a severe apneic phenotype in mice expressing *Phox2b^Δ8^* in the *Nkx2.2*‐derived progenitor domains, but not other embryological domains. While our findings show that RTN neurons and preBötC neurons are not embryologically derived from *Nkx2.2*, we observed *Nkx2.2^Cre^, Phox2b^Δ8^* mice possess cytoarchitectural defects throughout the VRC and is accompanied by disrupted inspiratory rhythmogenesis from the preBötC. Burst duration from the preBötC of *Nkx2.2^Cre^, Phox2b^Δ8^* mice is abbreviated, and the relationship between frequency and the variability of rhythmogenesis is changed. Furthermore, our inability to induce apneic phenotypes through chemogenetic silencing of *Nkx2.2*‐derived cells underscores that dysfunction in *Nkx2.2*‐derived neuronal networks alone does not lead to an apneic phenotype. These findings together support the notion that the cytoarchitectural defects in the VRC may be disrupting respiratory oscillators in the VRC via non‐cell autonomous developmental mechanisms. Such mechanisms cause selective loss of neurons, disorganized respiratory circuits, and likely contributes to the irregular breathing pattern and severe apneic phenotype.

Expiration is a passive process and becomes active during periods of increased metabolic demand. *Nkx2.2^Cre^, Phox2b^Δ8^* mutation, produces significant changes in expiration, increasing the expiratory time and decreasing the expiratory flow. An expiratory population of neurons has been described in the pFRG region (30). Evidences also suggest that the pFRG and chemosensitive neurons in the RTN may interact in a way that glutamatergic inputs from the RTN to the pFRG are necessary for the activation of the expiratory neurons during hypercapnia challenge, which is responsible for the generation of active expiratory pattern and the emergence of expiratory‐related bursts in sympathetic activity (70).

Loss of RTN/pFRG neurons likely contributes to a reduced central chemoreflex and increased apnea events in these animals, compromising ventilation. Recent studies have also identified a cluster of *Atoh‐1* derived cells in the rostral rhombic lip (rRL) contribute to respiratory pattern derived hypoxia (24). Furthermore, the absence of RTN/pFRG also compromise innervations to the ventral respiratory column, which may affect the control of respiratory pattern generators, as well as changing the air outflow. We also note that among brainstem nuclei affected in the *Nkx2.2^Cre^, Phox2b^Δ8^* mice, key structures known to modulate cardiac autonomic function undergo dysgenesis. Specifically, NA and DMNV have distinct populations of cardiac vagal preganglionic neurons, displaying crucial restraining modulation of heart rate (32, 38). As most neurons lost in *Nkx2.2^Cre^, Phox2b^Δ8^* DMNV, these likely included a spectrum of vagal cardiac preganglionic neurons. These neurons densely innervate the atria, sinoatrial and atrioventricular nodal tissue and also conducting tissue, controlling the heart rate, rate of atrioventricular conduction and the strength of atrial contraction (19). Vagal innervations also project to cardiac ventricles and atria (9). In summary, considering the importance of cardiac parasympathetic activity derived from the DMNV, we cannot exclude the possibility that progressive decline of cardiac function contributes to the decreased postnatal viability at birth in *Nkx2.2^Cre^, Phox2b^Δ8^* pups.

*Nkx2.2* has a well‐documented role in generating serotonergic neurons (20), as well as visceral motor neurons (42). *Nkx2.2^−/−^* pups breath normally at birth but die on P6 from pancreatic insufficiency (59). *Nkx2.2* has a primary role in ventral embryonic patterning (4), yet *Nkx2.2^−/−^* animals do not generate defects in *Phox2b*‐derived branchiomotor neurons, presumably through redundancy with *Nkx2.9* (31). Our intersectional genetic studies show unequivocally that nVII shows derivation from both *Phox2b* and *Nkx2.2* expressing cells. Compelling evidence in the scientific literature suggests that the location of nVII may be unimportant for proper RTN function. For instance, the human nVII nucleus is located at a position that is significantly more rostral and dorsal relative to the murine nVII. Two studies reported a cluster of PHOX2B‐positive neurons adjacent to human nVII considered as candidate human RTN neurons. Lavezzi and colleagues reported cells showing cytoplasmic PHOX2B immunoreactivity located between the nVII and the superior olivary nuclear complex (34). Rudzinski and Kapur studied human fetal brains, identifying a population of PHOX2B‐positive, TH‐negative cells dorsolateral to the human nVII at the level of the pontomedullary junction (53). The cells in Rudzinski and Kapur’s study show relatively close apposition to the pial surface, although a significant displacement away for the glial limitans exists for this population relative to their murine counterparts. The location of the putative human RTN proposed in these two studies is similar, but the Rudzinski and Kapur cell population is located significantly more ventrolateral to that described in the Lavezzi and colleagues’ study. Nevertheless, despite human nVII localization being different, it is presumed that human RTN still functions analogously to its murine RTN counterpart. Of note, data from the *reeler* and *scrambler* mouse models, both of which show marked neuroblast migration defects, also support a role for preserved respiratory rhythm generation despite a displaced nVII. Reeler mice suffer ataxia, tremors, and impaired motor coordination due to aberrant layer formation in neocortex, hippocampus, and cerebellum resulting from mutation in the *reeler* gene (11, 25). Scrambler mice demonstrate similar cytoarchitectural disorganization to Reeler mice (54), and suffer from mutations in the gene *disabled homologue 1 (Dab1),* which encodes the Dab1 adaptor protein (28, 54), a component of the REELER signal transduction system. Seminal work by Rossel, and colleagues demonstrated that in both reeler and scrambler mice, the nVII migrates laterally from the neuroepithelium but does not migrate to the normal ventral position (52). As these mice survive into adulthood, respiratory rhythm generation is likely preserved, but subtle defects in respiratory control have not been explored in these mice. In summary, data from comparative neuroanatomical analyses as well as mouse models of disease offer compelling evidence that proper nVII development, even if aberrantly migrated, correlates with the presence of a respiratory rhythm. In contrast, in our CCHS model, dysgenesis of visceral motor neurons such as nVII result in an apneic phenotype. We propose that this group of neurons may serve as developmental organizers for the proper generation of respiratory oscillators. For instance, *Nkx2.2*‐derived visceral motor neurons or glia could play an essential role in the trafficking and positioning of respiratory oscillator neurons during early embryogenic development.

### Molecular mechanisms of *Phox2b^Δ8^* induced developmental neuropathology

A potential limitation in our study is the fact that we do not identify GFP expression in the *Nkx2.2^cre^, Phox2b^Δ8^* animals. Our inducible *Phox2b^Δ8^* humanized mutation generates a bicistronic mRNA containing *Phox2b^Δ8^* coding region, an IRES sequence, and a *GFP* coding region. Identification of *Phox2b^Δ8^* is not possible by antibody‐mediated immunohistochemistry. As a positive control for GFP expression, we performed side‐by‐side analyses with *Olig3^Cre^, Phox2b^Δ8^* mice at P0/P1 which showed robust GFP expression in the dorsal rhombencephalon (Figure S11). These data indicate potentially that the *Phox2b^Δ8^*::*GFP* bicistronic transcript is silenced in *Nkx2.2^Cre^, Phox2b^Δ8^* animals. This raises a few interesting possibilities as to how *Phox2b* dysfunction may occur in CCHS. We predict that either *Phox2b^Δ8^* is expressed at very low levels with negligent transcriptional impact, or that *Phox2b^Δ8^* is expressed and causes transcriptional dysfunction due to its aberrant C‐terminus. During normal murine development, the ratio between murine *Phox2b* splice variant 201 and splice variant 202 is finely calibrated, with the 201/202 ratio increasing with developmental age. One mechanistic possibility to pursue is the notion that the ratio of 201:202 may be critical for proper neuron development. If this scenario was to be the cause, in *Nkx2.2^Cre^, Phox2b^Δ8^* animals, dysfunctional *Phox2b* splice variant 201‐dependent transcription, either because *Phox2b^Δ8^* is acting as a dysfunctional transcription factor or as an absent transcription factor, would alter the physiological 201:202 ratio. We proposed (Figure 8E) that the net effect would be an increase in ventral *Nkx2.2*‐derived cell proliferation at the expense of visceral motor neuron development. Our data point to the existence of a potential Phox2b+ transit amplifying progenitor stage located at the interface of the ventricular zone and the ISLET1‐positive marginal zone. In CCHS, there is a significant decrease in CldU incorporation in the Phox2b+ progenitor cells, thereby leading to a net decrease in the Islet1+ cells. We posit that maintaining a ratio of functional 201:202 *Phox2b* transcripts may be critical for proper cell cycle exit of pMNv progenitor cells so that they proceed into PHOX2B‐positive/ISLET‐1‐positive visceral motor neurons. By diminishing the Phox2b+ proliferative compartment, the visceral motor neuron pool does not develop properly. Thus, the deficits in visceral motor neurons noted at E18.5 in this CCHS model, including the NA, DMNV, and CNVII, are cell autonomous within the Nkx2.2 progenitor domain. However, the maldevelopment of these structures leads to defects in respiratory neural networks in cells not derived from the Nkx2.2 progenitor domain. These dysfunctions in respiration which mediate the majority of the CCHS phenotype are non‐cell autonomous.

## Conflict of Interest

The authors declare that they have no conflict of interest.

## Supporting information


**Figure S1.** Justification for pooling of control genotypes.

**Figure S2.** Respiratory physiology of *Olig3^Cre^*, Phox2b^Δ8^ mice.

**Figure S3.** Hyperoxic hypercapnic challenge in *Olig^Cre^*, Phox2b^Δ8^ mice.

**Figure S4.** F, TV, and MV analysis following chemogenetic silencing of *Nkx2.2*‐derived cells.

**Figure S5.** Ti, Te, PIF, PEF, and EF50 analysis following chemogenetic silencing of *Nkx2.2*‐derived cells.

**Figure S6.** PAU, PENH, EEP, EIP analysis following chemogenetic silencing of *Nkx2.2*‐derived cells.

**Figure S7.** Respiratory function analysis following chemogenetic silencing of *Nkx2.2*‐derived circuits in newborn mice.

**Figure S8.** Neuropathological findings in *Olig3^Cre^*, Phox2b^Δ8^ mice.

**Figure S9.** Cardiovascular physiology of *Olig3^Cre^*, Phox2b^Δ8^ mice.

**Figure S10.** Machine learning analysis of cell cycle data.

**Figure S11.** GFP expression analysis in *Nkx2.2^cre^* and *Olig3^Cre^* drivers.

**Table S1.** Genotyping primers.
**Table S2.** Antibodies.
**Table S3.** Oligonucleotide sequences used for RNA FISH probes.
**Table S4.** The linear model was performed in Rstudio using the model lm(f ~ Ti+Te).
**Table S5.** VO_2_ linear regression model summary.
**Table S6.** VCO_2_ linear regression model summary.
**Table S7.** Energy expenditure linear regression model summary.
**Table S8.** Activity linear regression model summary.
**Table S9.** Quantification of Phox2b and Islet1 positive cell on the ventral neuroepithlium.

Supplementary Material

## Data Availability

Derived data supporting the findings of this study are available from the corresponding author on request.
