# Supplementary material for: Neonatal apneic phenotype in a murine congenital central hypoventilation syndrome model is induced through non‐cell autonomous developmental mechanisms
Source: Brain Pathol. 2020 Aug 4;31(1):84–102. doi: 10.1111/bpa.12877 (PMC7881415; doi:10.1111/bpa.12877)
Supplement: Supplementary file 1 — Figure S1. Justification for pooling of control genotypes. [file BPA-31-84-s008.pdf]

Alzate, Liu, et al., Supplemental Figure 1

A

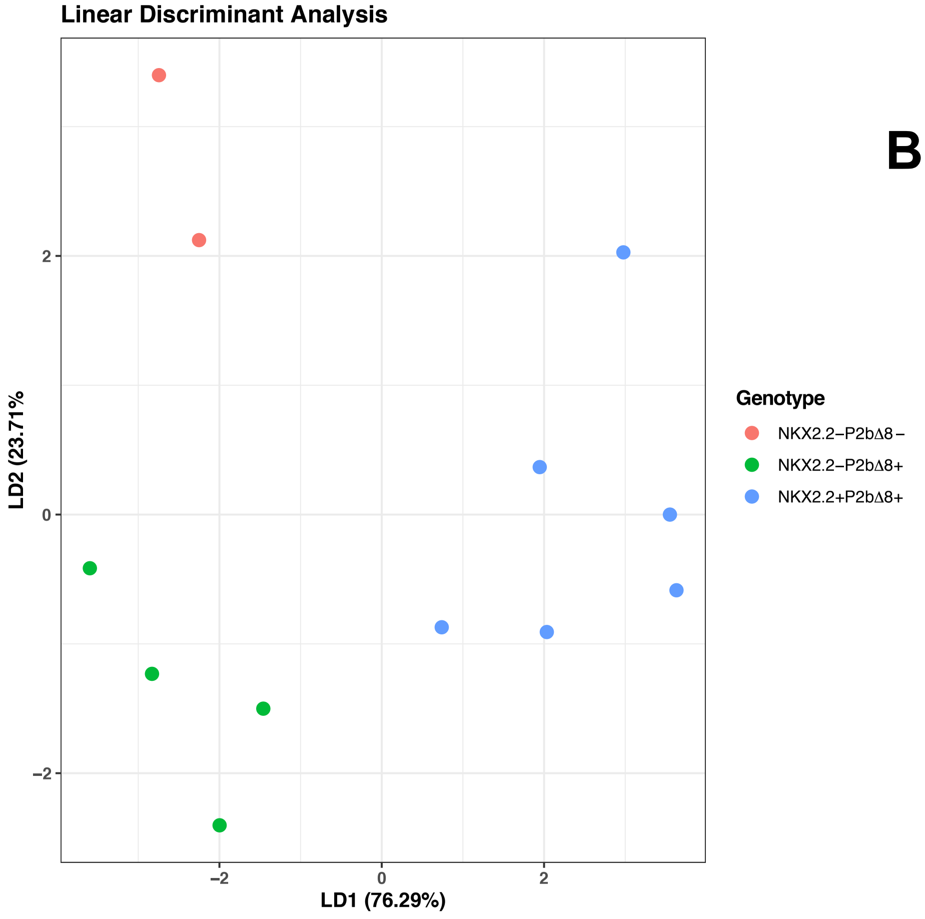

B **Machine Learning Prediction Trained on data excluding NKX2.2-P2bΔ8- data**

Reference Genotype	Predicted Genotype, RF Model	Predicted Genotype, SVM Model	Predicted Genotype, KNN Model	Predicted Genotype, LDA Model
Control NKX2.2-P2bΔ8-	Control NKX2.2-P2bΔ8+	Control NKX2.2-P2bΔ8+	Control NKX2.2-P2bΔ8+	Control NKX2.2-P2bΔ8+
Control NKX2.2-P2bΔ8-	Control NKX2.2-P2bΔ8+	Control NKX2.2-P2bΔ8+	Control NKX2.2-P2bΔ8+	Control NKX2.2-P2bΔ8+
