# Supplementary material for: Neonatal apneic phenotype in a murine congenital central hypoventilation syndrome model is induced through non‐cell autonomous developmental mechanisms
Source: Brain Pathol. 2020 Aug 4;31(1):84–102. doi: 10.1111/bpa.12877 (PMC7881415; doi:10.1111/bpa.12877)
Supplement: Supplementary file 2 — Figure S2. Respiratory physiology of Olig3Cre, Phox2bΔ8 mice. [file BPA-31-84-s009.pdf]

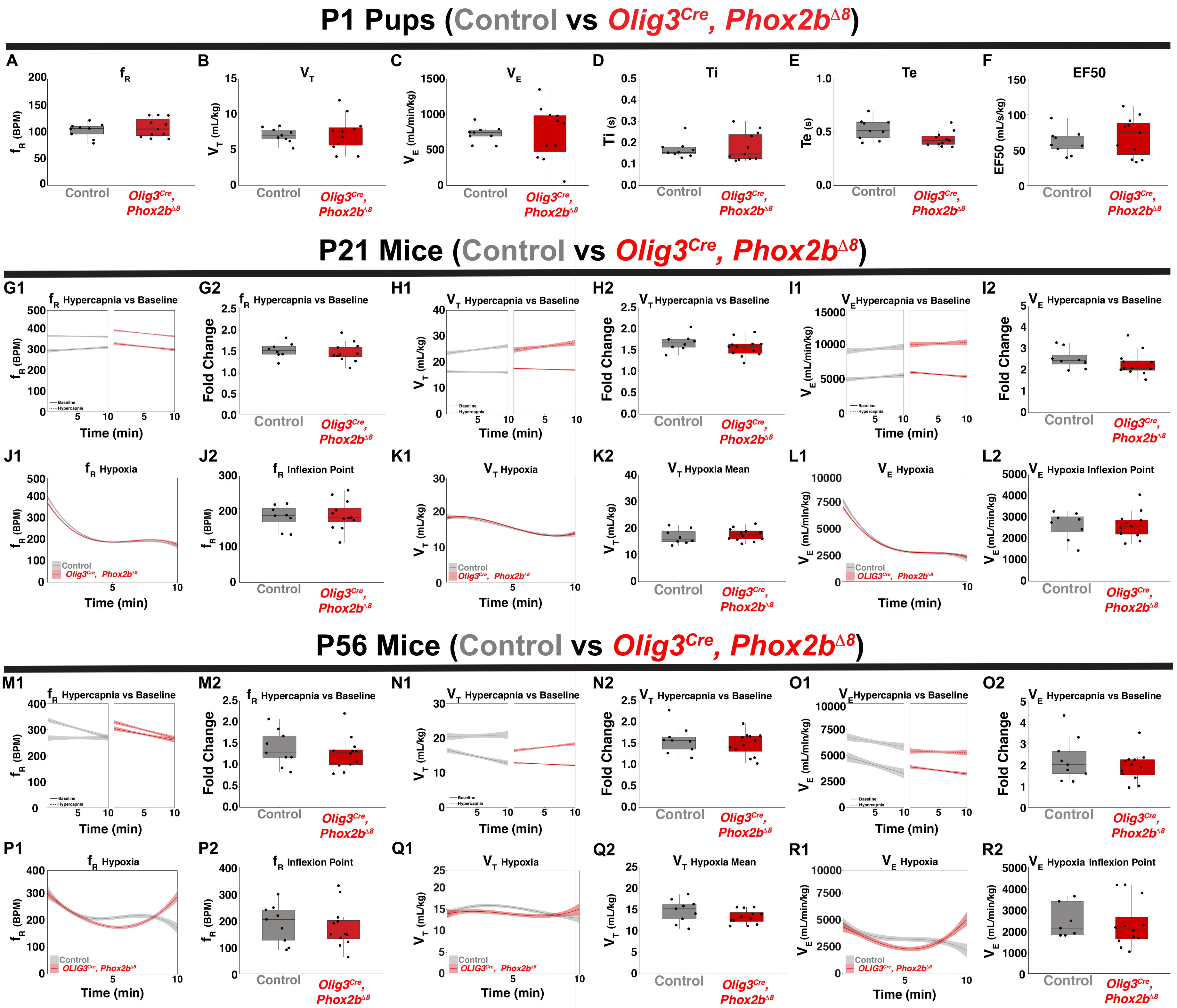
P1 Pups (Control vs Olig3Cre, Phox2bΔ8)
A fR
B VT
C VE
D Ti
E Te
F EF50
P21 Mice (Control vs Olig3Cre, Phox2bΔ8)
G1 fR Hypercapnia vs Baseline
G2 fR Hypercapnia vs Baseline
H1 VT Hypercapnia vs Baseline
H2 VT Hypercapnia vs Baseline
I1 VE Hypercapnia vs Baseline
I2 VE Hypercapnia vs Baseline
J1 fR Hypoxia
J2 fR Inflexion Point
K1 VT Hypoxia
K2 VT Hypoxia Mean
L1 VE Hypoxia
L2 VE Hypoxia Inflexion Point
P56 Mice (Control vs Olig3Cre, Phox2bΔ8)
M1 fR Hypercapnia vs Baseline
M2 fR Hypercapnia vs Baseline
N1 VT Hypercapnia vs Baseline
N2 VT Hypercapnia vs Baseline
O1 VE Hypercapnia vs Baseline
O2 VE Hypercapnia vs Baseline
P1 fR Hypoxia
P2 fR Inflexion Point
Q1 VT Hypoxia
Q2 VT Hypoxia Mean
R1 VE Hypoxia
R2 VE Hypoxia Inflexion Point
Control
Olig3Cre, Phox2bΔ8
Baseline
Hypercapnia
Time (min)
Fold Change
fR (BPM)
VT (mL/kg)
VE (mL/min/kg)
Ti (s)
Te (s)
EF50 (mL/s/kg)
