# Supplementary figures and images for: Neonatal apneic phenotype in a murine congenital central hypoventilation syndrome model is induced through non‐cell autonomous developmental mechanisms
Source: Brain Pathol. 2020 Aug 4;31(1):84–102. doi: 10.1111/bpa.12877 (PMC7881415; doi:10.1111/bpa.12877)

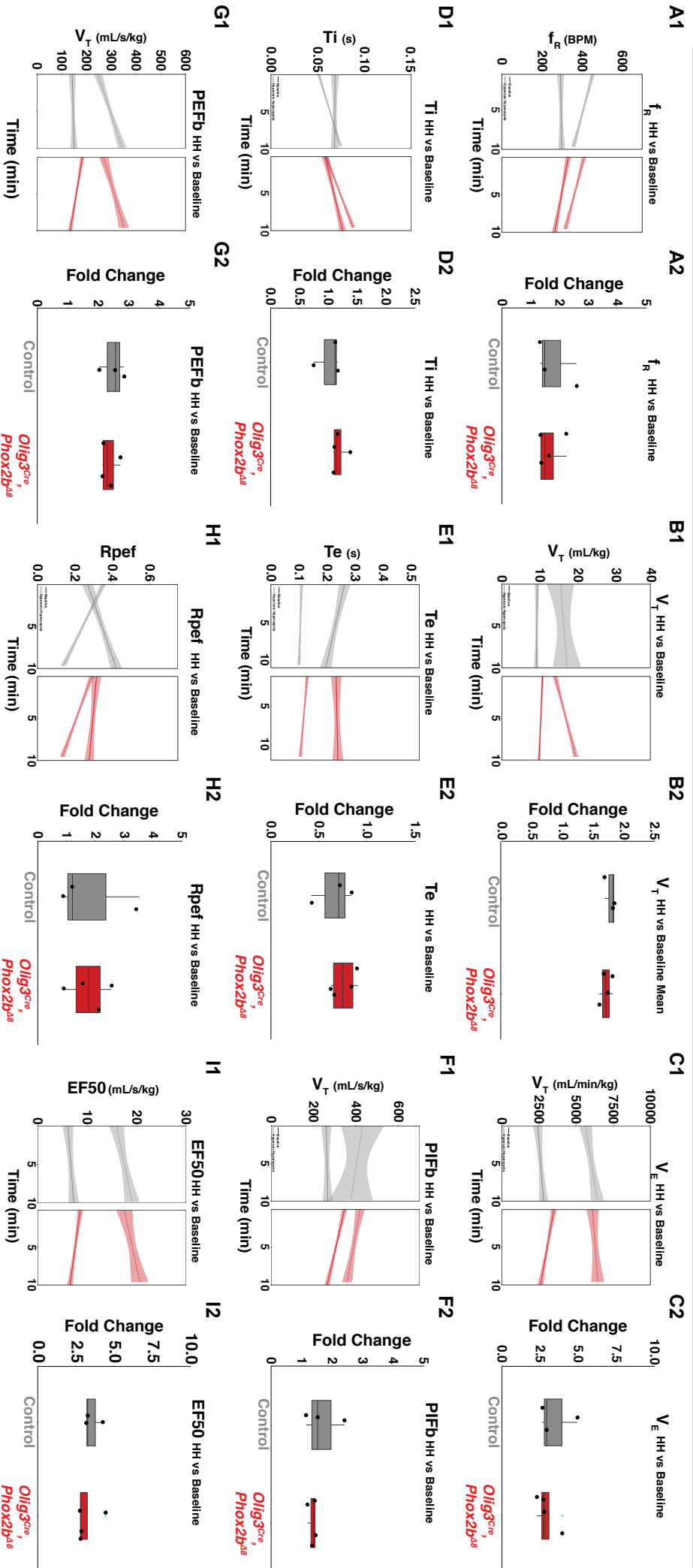

Supplement: Supplementary file 3 — Figure S3. Hyperoxic hypercapnic challenge in OligCre, Phox2bΔ8 mice. [file BPA-31-84-s011.pdf]

Liu, Alzate, et al., Supplemental Figure 4

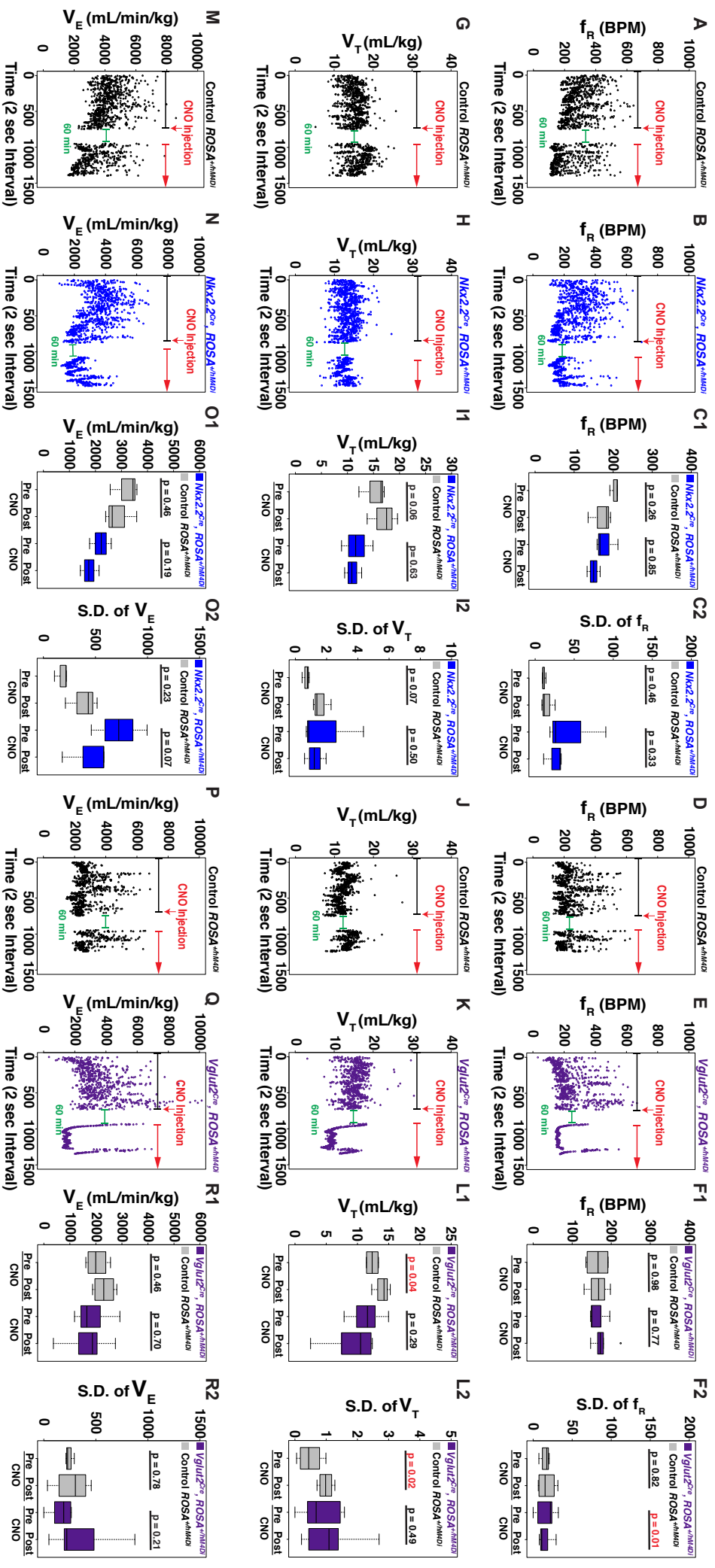

Supplement: Supplementary file 4 — Figure S4. F, TV, and MV analysis following chemogenetic silencing of Nkx2.2‐derived cells. [file BPA-31-84-s001.pdf]

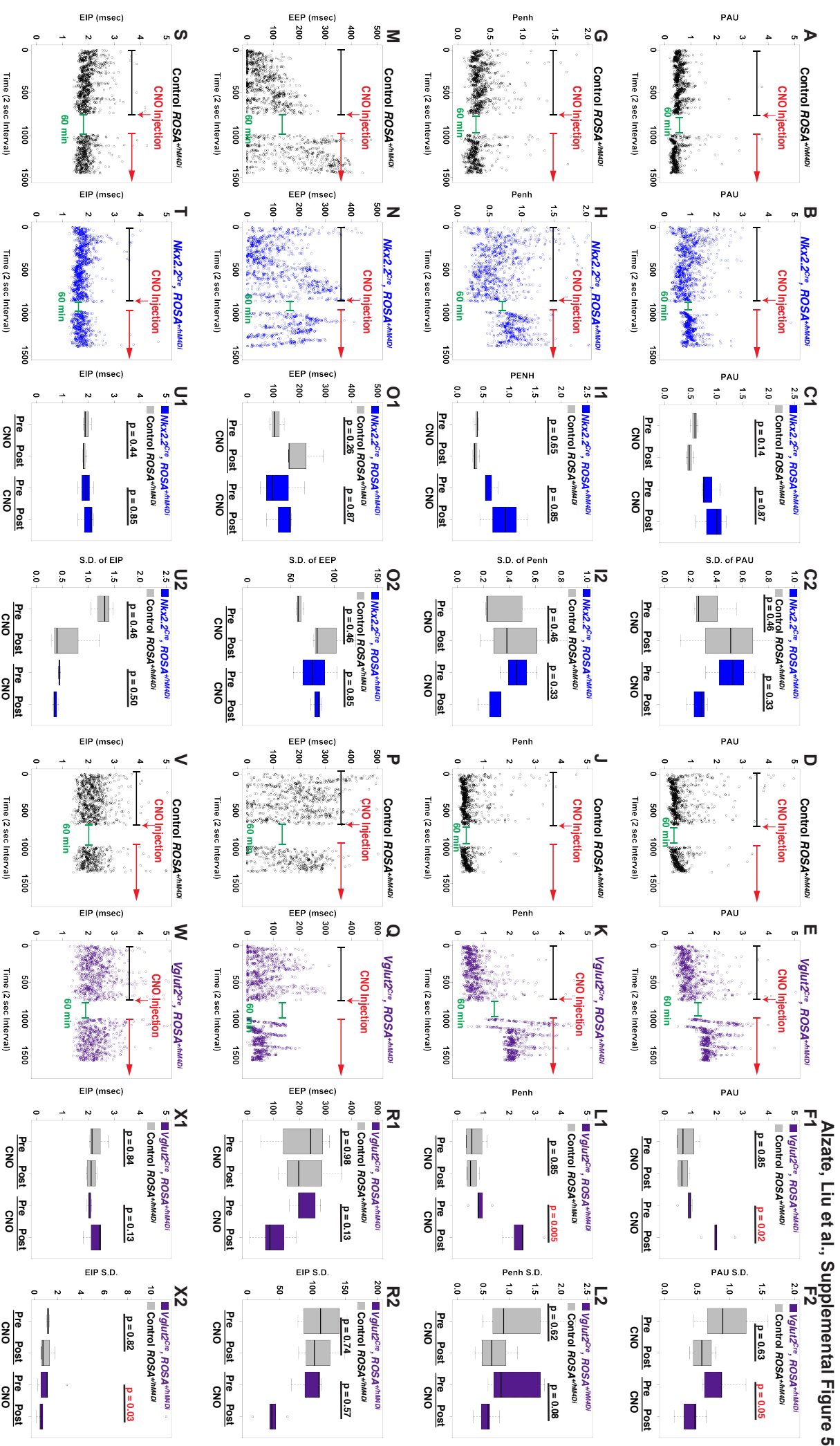

Supplement: Supplementary file 5 — Figure S5. Ti, Te, PIF, PEF, and EF50 analysis following chemogenetic silencing of Nkx2.2‐derived cells. [file BPA-31-84-s002.pdf]

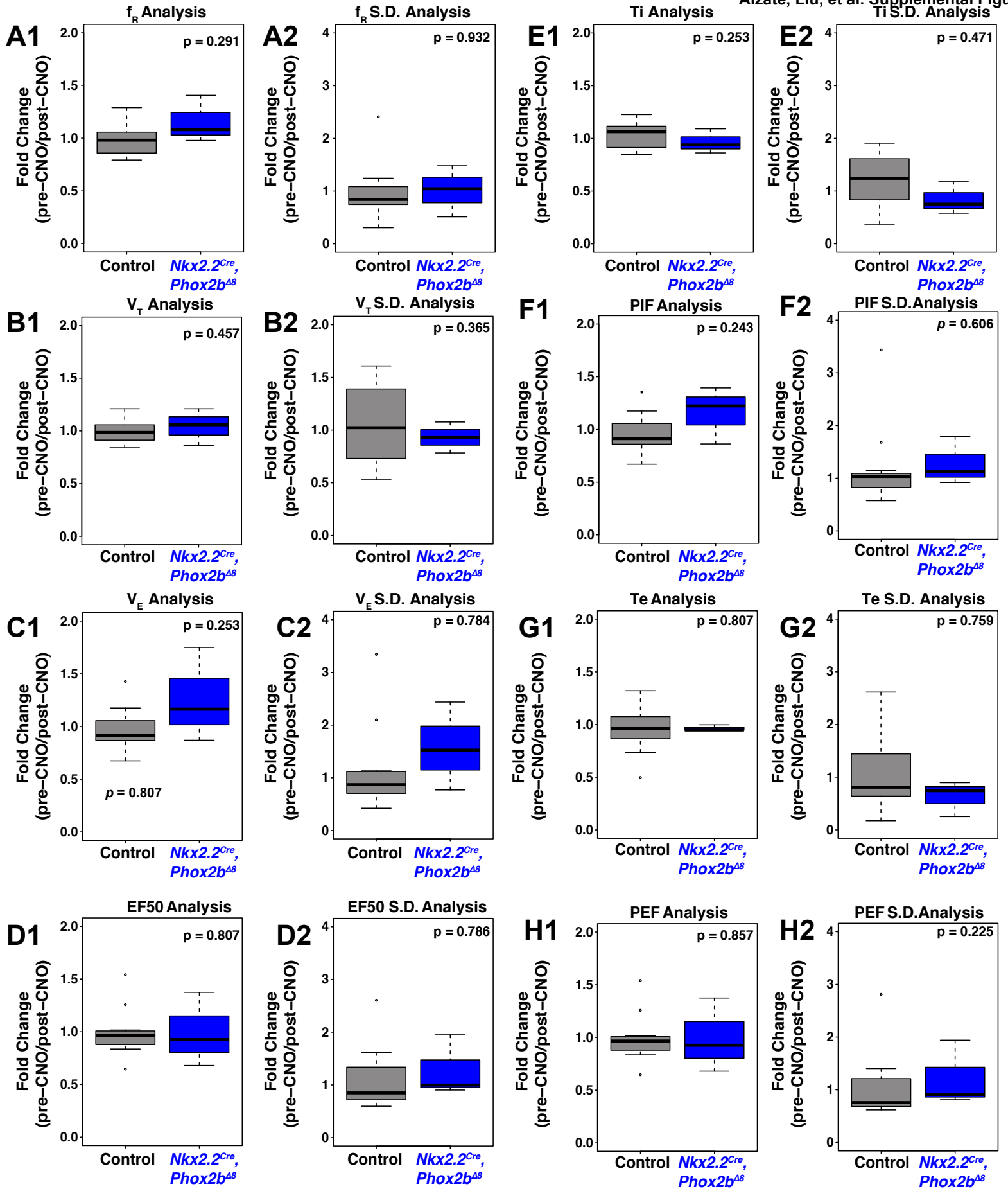

Supplement: Supplementary file 7 — Figure S7. Respiratory function analysis following chemogenetic silencing of Nkx2.2‐derived circuits in newborn mice. [file BPA-31-84-s004.pdf]

Alzate, Liu, et al., Supplemental Figure 8

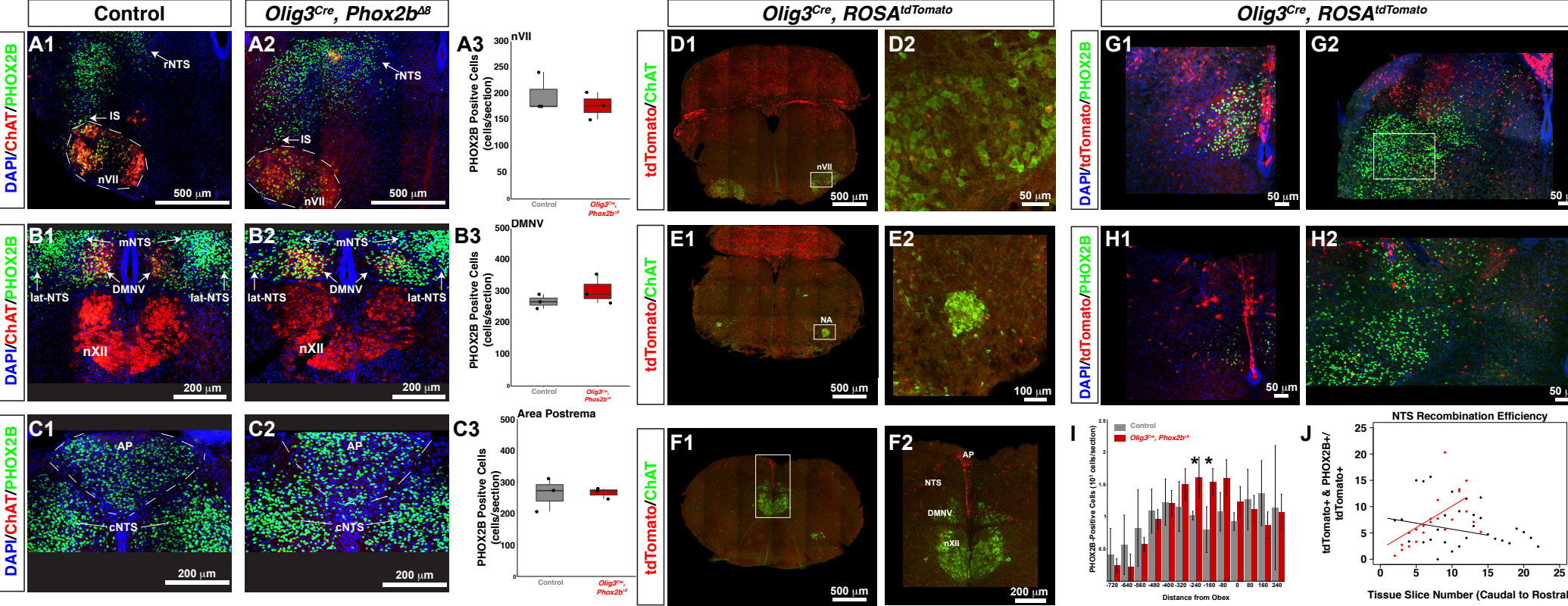

Supplement: Supplementary file 8 — Figure S8. Neuropathological findings in Olig3Cre, Phox2bΔ8 mice. [file BPA-31-84-s005.pdf]

# Alzate, Liu, et al., Supplementary Figure 9

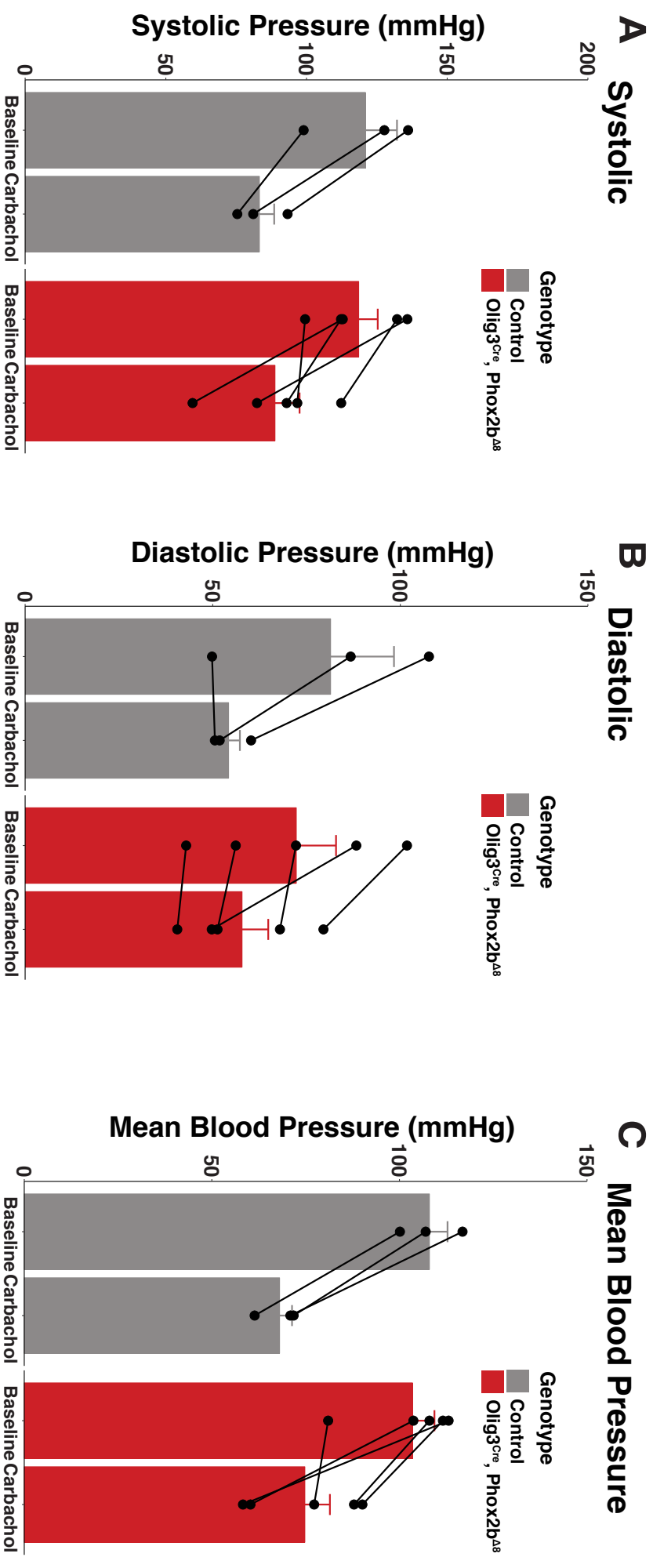

Supplement: Supplementary file 9 — Figure S9. Cardiovascular physiology of Olig3Cre, Phox2bΔ8 mice. [file BPA-31-84-s006.pdf]

# Alzate, Liu, et al., Supplemental Figure 11

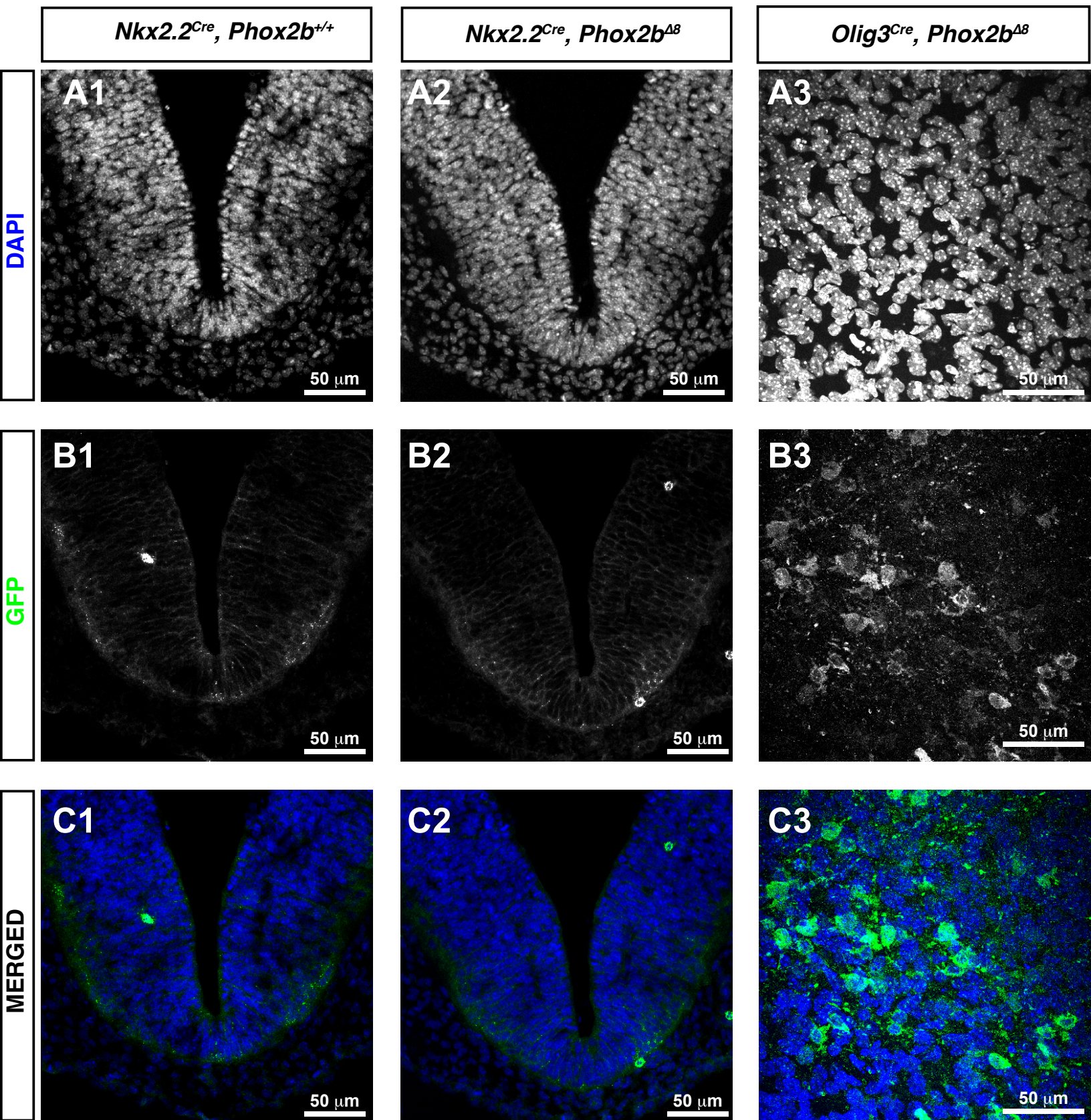

Supplement: Supplementary file 11 — Figure S11. GFP expression analysis in Nkx2.2cre and Olig3Cre drivers. [file BPA-31-84-s012.pdf]
