# Supplementary material for: Neonatal apneic phenotype in a murine congenital central hypoventilation syndrome model is induced through non‐cell autonomous developmental mechanisms
Source: Brain Pathol. 2020 Aug 4;31(1):84–102. doi: 10.1111/bpa.12877 (PMC7881415; doi:10.1111/bpa.12877)

Alzate, Liu, Jonnes, Supplementary Figure zzzz

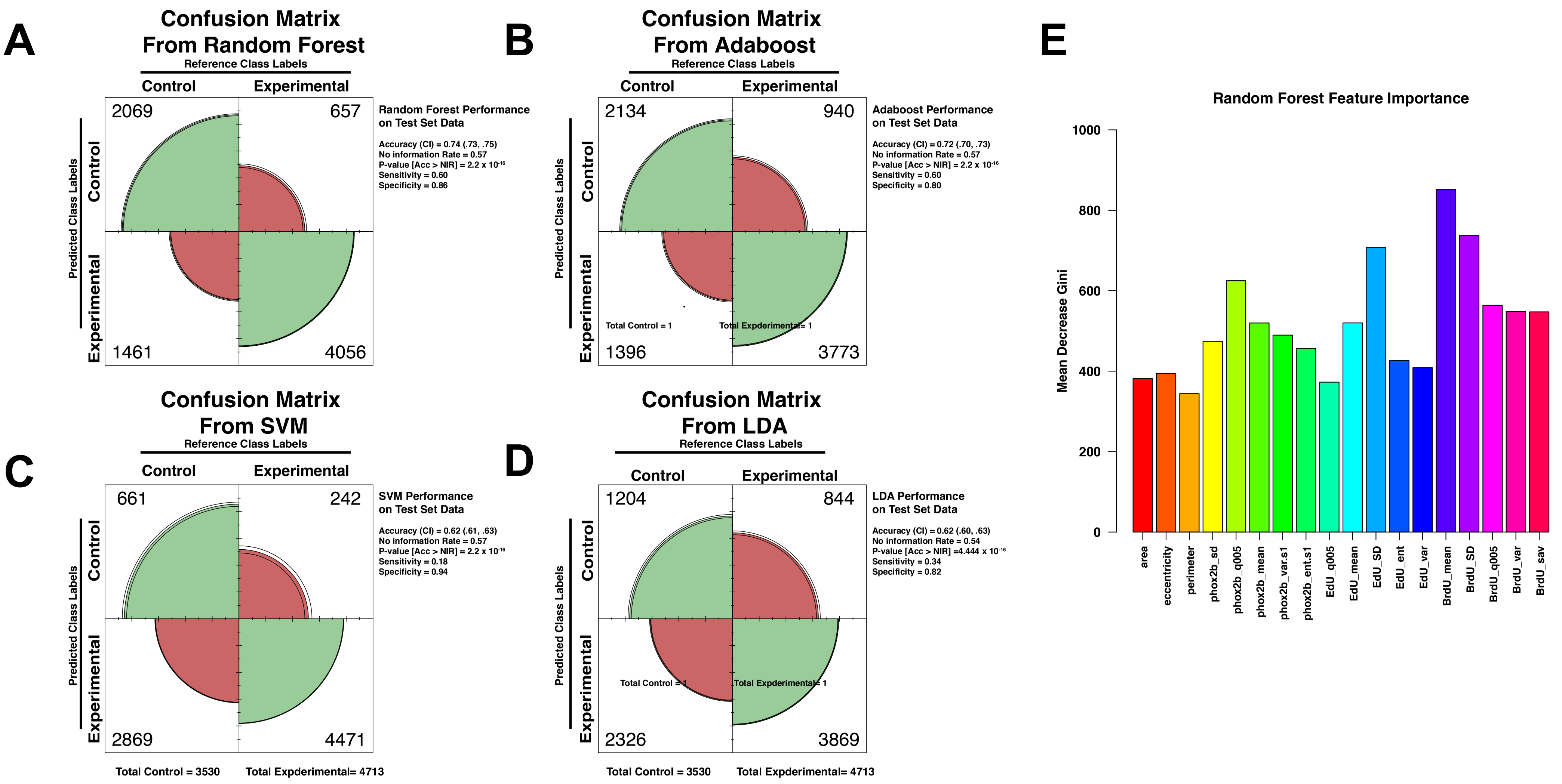

Supplement: Supplementary file 10 — Figure S10. Machine learning analysis of cell cycle data. [file BPA-31-84-s007.pdf]
