# Supplementary material for: Neonatal apneic phenotype in a murine congenital central hypoventilation syndrome model is induced through non‐cell autonomous developmental mechanisms
Source: Brain Pathol. 2020 Aug 4;31(1):84–102. doi: 10.1111/bpa.12877 (PMC7881415; doi:10.1111/bpa.12877)
Supplement: Supplementary file 12 — Table S1. Genotyping primers. Table S2. Antibodies. Table S3. Oligonucleotide sequences used for RNA FISH probes. Table S4. The linear model was performed in Rstudio using the model lm(f ~ Ti+Te). Table S5. VO2 linear regression model summary. Table S6. VCO2 linear regression model summary. Table S7. Energy expenditure linear regression model summary. Table S8. Activity linear regression model summary. Table S9. Quantification of Phox2b and Islet1 positive cell on the ventral neuroepithlium. [file BPA-31-84-s013.docx]

**Supplementary Table 1: Genotyping Primers**

| **Mouse Line** | **Strain #** | **Strain Name** | **Primers** | **Band Sizes** | **Reference** | **Obtained from** |
| --- | --- | --- | --- | --- | --- | --- |
| *Atoh1^Cre^* | Jax: 011104 | B6.Cg-Tg(Atoh1-cre)1Bfri/J | **Tg FWD**  5'-CCG GCA GAG TTT ACA GAA GC-3' | Tg = 450 bp | (3) | Jackson Laboratories |
|  |  |  | **Tg REV** 5'-ATG TTT AGC TGG CCC AAA TG-3' | CTR = 324 bp |  |  |
|  |  |  | **CTR FWD** 5'-CTA GGC CAC AGA ATT GAA AGA TCT-3' |  |  |  |
|  |  |  | **CTR REV** 5'-GTA GGT GGA AAT TCT AGC ATC ATC C-3' |  |  |  |
| *Nkx2.2^Cre^* | MGI: 5290102 | Tg(Nkx2-2*-cre)1Mtse | **FWD** 5'-CGT TCA CCG GCA TCA ACG TTT-3' | 232 bp | (8) | Michael Matise, Rutgers Robert Wood Johnson Medical School |
|  |  |  | **REV** 5'-GCG GCA TGG TGC AAG TTG AAT-3' |  |  |  |
| *Olig3^Cre^* | MGI: 3841452 | Olig3^tm1(cre)Ynka^ | **Olig3Cre-S** 5'-TTC AGG CAC ACA CCA ATG TGC-3' | 300 bp | (7) | Yasushi Nakagawa, University of Minnesota |
|  |  |  | **Olig3Cre-AS** 5'-ATC ACT CGT TGC ATC GAC CGG-3' |  |  |  |
| *Phox2b^Δ8^* | Jax: 025436 | B6.129(Cg)-Phox2btm1Rth/J | **FWD** 5'-GCC CAC AGT GCC TCT TAA CTC-3' | Mutant = 450 bp | (4) | David Rowitch, UCSF |
|  |  |  | **REV** 5'-CGT ACT CTT AAA CGG GCG TCT C-3' | Wild type = 334 bp |  |  |
| *ROSA^TdTomato^* | Jax: 007914 | B6.Cg-Gt(ROSA)26Sor^tm14(CAG-tdTomato)Hze^/J | **WT FWD** 5'-AAG GGA GCT GCA GTG GAG TA-3' | Mutant = 196 bp | (2) | Jackson Laboratories |
|  |  |  | **WT REV** 5'-CCG AAA ATC TGT GGG AAG TC-3' | Wild type = 297 bp |  |  |
|  |  |  | **MUT FWD** 5'-GGC ATT AAA GCA GCG TAT CC-3' |  |  |  |
|  |  |  | **MUT REV** 5'-CTG TTC CTG TAC GGC ATG G-3' |  |  |  |
| *Vglut2^Cre^* | Jax: 016963 | Slc17a6^tm2(cre)Lowl^/J | **FWD** 5'-CGG TAC CAC CAA ATC TTA CGG-3' | Mutant = ~ 850 bp | (6) | Jackson Laboratories |
|  |  |  | **WT REV** 5'-CAT GGT CTG TTT TGA ATT CAG-3' | Wild type = 299 bp |  |  |
|  |  |  | **MUT REV** 5'-ATC GAC CGG TAA TGC AGG CAA-3' |  |  |  |
| *ROSA^+/hM4Di^* | Jax: 026219 | B6N.129-Gt(ROSA)26Sor^tm1(CAG-CHRM4*,-mCitrine)Ute^/J | **MUT REV** 5'-TCA TAG CGA TTG TGG GAT GA-3' | Mutant = 200 bp | (9) | Jackson Laboratories |
|  |  |  | **MUT FWD** 5'-CGA AGT TAT TAG GTC CCT CGA C-3' | Wild type = 297 bp |  |  |
|  |  |  | **WT FWD** 5'-AAG GGA GCT GCA GTG GAG TA-3' |  |  |  |
|  |  |  | **WT REV** 5'-CCG AAA ATC TGT GGG AAG TC-3' |  |  |  |
| Phox2b-FLPo | JAX: 022407 | B6;D2-Tg(Phox2b-flpo)3276Grds/J | **18582-FW**DGAG CTT CGA CAT CGT GAA CA | Tg = 330 bp | (1) | Jackson Laboratories |
|  |  |  | **18583-REV**ACA GGG TCT TGG TCT TG | Control = 200bp |  |  |
|  |  |  | **oIMR8744-S** 5'-CAA ATG TTG CTT GTC TGG TG-3' |  |  |  |
|  |  |  | **oIMR8745-AS** 5'-GTC AGT CGA GTG CAC AGT TT-3' |  |  |  |
| Intersectional RC::FLTG | JAX 026932 | B6.Cg-Gt(ROSA)26Sor^tm1.3(CAG-tdTomato,-EGFP)Pjen^/J | **24009-MUT 5'-**FWDGCA ACG TGC TGG TTA TTG TG-3' | Tg= 160 bp | (5) | Jackson Laboratories |
|  |  |  | **23801-MUT 5'-**REVTAT TTG GTG TCC CCG AGA AG-3' | Control = 297 bp |  |  |
|  |  |  | **oIMR9020-WT 5'-**FWDAAG GGA GCT GCA GTG GAG TA-3' |  |  |  |
|  |  |  | **oIMR9021-WT 5'-**REVCCG AAA ATC TGT GGG AAG TC-3' |  |  |  |

Supplementary Table 2. Antibodies

| **Antigen** | **Description of Immunogen** | **Source, host species, catalog No.** | **Concentration** | **RRID** |
| --- | --- | --- | --- | --- |
| Anti-ChAT | Human placental enzyme | Millipore, goat, AB144P | 1:300 | AB_2079751 |
| Anti-Islet1 | Recombinant C-terminal of rat Islet-1 (aa 178-349) | Developmental Studies Hybridoma Bank, mouse IgG1, 40.2D6 | 1:20 | AB_528315 |
| Anti-NK1R | Synthetic peptide corresponding to a 23-amino acid sequence (385-407) of the C-terminal region of Rat Substance P Receptor (NK-1) | Millipore, rabbit, AB5060 | 1:500 | AB_2200636 |
| Anti-Nkx2.2 (Abcam) | Recombinant fragment within Human Nkx2.2 aa 1-150 (N terminal). | Abcam, rabbit, ab191077 | 1:200 | Not available |
| Anti-Nkx2.2 (DSHB) | Nkx2.2-GST fusion protein expressed in E. coli. | Developmental Studies Hybridoma Bank, mouse IgG2B, 74.545 | 1:3 | AB_531794 |
| Anti-Phox2B (C-terminal) | Synthetic peptide corresponding to the C-terminal region of Human PHOX2B (250-C-terminus) | Abcam, rabbit, AB183741 | 1:500 | Not available |
| Anti-Phox2B (N-terminal) | Raised against amino acids 11-70 mapping near the N-terminus of Phox2b of human origin | Santa Cruz, mouse IgG2A, SC-376993 | 1:250 | Not available |
| Anti-TryptH | Recombinant rabbit tryptophan hydroxylase | Sigma, mouse IgG3, T0678 | 1:250 | AB_261587 |
| Anti-TH | Denatured tyrosine hydroxylase from rat pheochromocytoma | Millipore, rabbit, ab152 | 1:500 | AB_390204 |
| Anti-VGlut2 | Synthetic peptide corresponding to the C-terminal region of Mouse VGLUT2 conjugated to KLH (NP_543129.3) | Abcam, rabbit, AB84103 | 1:100 | AB_10674784 |

Supplementary Table 3. Oligonucleotide Sequences Used for RNA FISH Probes.

| **mPhox2b Variant 1 probe list** | | **mPhox2b Variant 2 probe list** | |
| --- | --- | --- | --- |
| **Probe** | **Sequence** | **Probe** | **Sequence** |
| 1 | AGAATCTGGGATGGAGGTGA | 1 | TTTGGAACCGTAAGCGTCAG |
| 2 | CGAAGATAAGACGCTGGCGA | 2 | GTGCGCATAACACTCCAAAT |
| 3 | ATACTGCTCTTCACTAAGGC | 3 | AATTACCTAGAGTCTGTGTC |
| 4 | GAATCGCGATCATTTTAGGC | 4 | TGCTTTGGTGGGAAACACTG |
| 5 | ACGTCATTTTCTCGTTGTTT | 5 | TTCGTTCAAACTCCCAAACC |
| 6 | CTGAGACAGACATACACCCG | 6 | ACTTCTCTCCAAAAAAGCCT |
| 7 | TCACTGAGTTGGTTGTGGTG | 7 | GATGCAATCTTCCTCTCACG |
| 8 | CGTCTCCTTTGTATTTTCAA | 8 | GCTGACATAGATACAGGTAG |
| 9 | GTTCGCAAGACCAAAACCTC | 9 | GGCAAACGAATCACGCAATT |
| 10 | CAGTCGTGAGCGAGAGAATT | 10 | GCTTGAGTTAAGAGGCACTG |
| 11 | TATAGAGGAGCCGCAAGACA | 11 | GGGCACTAGATTTTTTAAGG |
| 12 | GACAGGCATCAGTACTCTTT | 12 | AGGAAATTACTCCTTGCGCC |
| 13 | TGAGCCATCACTGCAATGAC | 13 | TAACAACCCCTTCGTGAATC |
| 14 | AAGCAGAGCTCCAGAAAGTC | 14 | CGGGTTTCTGACTCTAATGG |
| 15 | TAATGGCGAGATGGTTTCCA | 15 | CCCAATTGGTTAGCTATAGT |
| 16 | GAGTGTGGGAAATCAGTTGC | 16 | AGCTCCAGAAAGCACCTTTG |
| 17 | TGCGAAGATTAGGGTTCAGG | 17 | CACAGAGGAACCTTCTTAGT |
| 18 | TGAGAAGGGATCTCCTGTAC | 18 | TTCGCTTTGGTGACACAACG |
| 19 | ACATGAGACACGGTTTGCAG | 19 | TAATGGTGGAAGGAGGGTGC |
| 20 | GGGTGGCGGGAGAGAAAAAA | 20 | CTACTTCTAGGCTTCTCAGG |
| 21 | ACTCCAATGCAAAGCAGTGC | 21 | TACTCTTAAACGGGCGTCTC |
| 22 | CCAGCTATGTGGACTACAAC |  |  |
| 23 | GCTGGGTAAGACTGATTTCT |  |  |
| 24 | GATCTGAGACACTTTCTGCA |  |  |
| 25 | AGCTCGGATCACTCAATCAG |  |  |
| 26 | CAATATACAGTCTGTCCACC |  |  |
| 27 | GAGTCACAGAACACAGCTGT |  |  |
| 28 | CGGTCTGTACTGAGAGTAAC |  |  |
| 29 | TTATACGGTCACGTAGAGGA |  |  |
| 30 | GGAATCGAACCCGTGAGAAC |  |  |
| 31 | CTGAACCTGGTCTCTCTAAA |  |  |
| 32 | AGACAGACATGCACCTGATA |  |  |
| 33 | TGTTCACAAACATAGTCCCA |  |  |
| 34 | CATGTAGAAATATTTCCCCT |  |  |

**Supplementary Table 4:**

| **Genotype** | **Intercept** | **Intercept** | **Ti Coefficient** | **Ti *p*** | **Te coefficient** | **Te *p*** | **Adjusted R-squared** |
| --- | --- | --- | --- | --- | --- | --- | --- |
| Control | 186.8 | .000116 | -103.12 | 0.011 | -109.21 | 0.013 | 0.9727 |
| *NKX2.2^Cre^, PHOX2B^Δ8^* | 136.689 | 0.011 | -138.4 | 0.26 | -35.04 | 0.0245 | 0.7853 |

The linear model was performed in Rstudio using the model lm(f ~ Ti+Te). The significance of the control and mutant models were p = 0.002095 and p = 0.04624. Data was generated from taking the mean value from 6 mutant and 6 control pups. The difference in the Te coefficient is 73.96 units between the mutant and the control group. We tested if this difference between the Te coefficiences of control and mutants was statistically significance by permutation analysis in Rstudio. The permutation analysis demonstrated a probability of *p* = 0.039, indicating that this difference between control and mutant is unlikely due to chance.

**Supplementary Table 5: VO_2_ linear regression model summary**

## Call:

## lm(formula = VO2 ~ DARK * X4C * MUTANT * FED * MALE, data = clams_data)

##

## Residuals:

## Min 1Q Median 3Q Max

## -6177.9 -505.9 56.3 610.7 2717.3

##

## Coefficients:

## Estimate Std. Error t value Pr(>|t|)

## (Intercept) 3272.891 55.273 59.213 < 2e-16 ***

## DARK 428.319 78.109 5.484 4.29e-08 ***

## X4C 2524.304 97.887 25.788 < 2e-16 ***

## MUTANT 75.583 77.533 0.975 0.329664

## FED 742.464 78.469 9.462 < 2e-16 ***

## MALE -509.174 96.833 -5.258 1.49e-07 ***

## DARK:X4C -628.596 137.802 -4.562 5.15e-06 ***

## DARK:MUTANT -66.164 109.449 -0.605 0.545515

## X4C:MUTANT 362.134 128.744 2.813 0.004922 **

## DARK:FED -273.783 110.676 -2.474 0.013390 *

## X4C:FED 38.499 138.913 0.277 0.781678

## MUTANT:FED 3.778 109.553 0.034 0.972494

## DARK:MALE -259.010 137.055 -1.890 0.058816 .

## X4C:MALE -364.949 149.910 -2.434 0.014935 *

## MUTANT:MALE -45.158 120.374 -0.375 0.707559

## FED:MALE -442.528 138.016 -3.206 0.001349 **

## DARK:X4C:MUTANT -167.772 181.067 -0.927 0.354174

## DARK:X4C:FED 638.528 195.223 3.271 0.001077 **

## DARK:MUTANT:FED 49.123 154.773 0.317 0.750956

## X4C:MUTANT:FED -361.080 182.205 -1.982 0.047542 *

## DARK:X4C:MALE 385.253 211.101 1.825 0.068041 .

## DARK:MUTANT:MALE 305.612 170.307 1.794 0.072772 .

## X4C:MUTANT:MALE -580.729 187.516 -3.097 0.001962 **

## DARK:FED:MALE 215.336 194.482 1.107 0.268227

## X4C:FED:MALE 139.749 213.003 0.656 0.511783

## MUTANT:FED:MALE 259.234 171.365 1.513 0.130378

## DARK:X4C:MUTANT:FED 539.776 256.166 2.107 0.035136 *

## DARK:X4C:MUTANT:MALE -877.939 264.066 -3.325 0.000889 ***

## DARK:X4C:FED:MALE -20.741 299.252 -0.069 0.944744

## DARK:MUTANT:FED:MALE -155.723 241.564 -0.645 0.519173

## X4C:MUTANT:FED:MALE 631.369 266.420 2.370 0.017819 *

## DARK:X4C:MUTANT:FED:MALE 55.277 374.299 0.148 0.882598

**Supplementary Table 6: VCO_2_ linear regression model summary.**

## Call:

## lm(formula = VCO2 ~ DARK * X4C * MUTANT * FED * MALE, data = clams_data)

##

## Residuals:

## Min 1Q Median 3Q Max

## -4830.7 -493.2 96.8 565.6 2981.3

##

## Coefficients:

## Estimate Std. Error t value Pr(>|t|)

## (Intercept) 2630.195 47.123 55.816 < 2e-16 ***

## DARK 114.193 66.591 1.715 0.08641 .

## X4C 1916.922 83.453 22.970 < 2e-16 ***

## MUTANT 2.973 66.100 0.045 0.96413

## FED 1003.336 66.898 14.998 < 2e-16 ***

## MALE -410.572 82.555 -4.973 6.71e-07 ***

## DARK:X4C -458.874 117.482 -3.906 9.46e-05 ***

## DARK:MUTANT 25.203 93.310 0.270 0.78709

## X4C:MUTANT 319.589 109.759 2.912 0.00360 **

## DARK:FED 147.621 94.356 1.565 0.11773

## X4C:FED 639.692 118.430 5.401 6.79e-08 ***

## MUTANT:FED 35.145 93.399 0.376 0.70671

## DARK:MALE -106.841 116.845 -0.914 0.36054

## X4C:MALE -375.565 127.805 -2.939 0.00331 **

## MUTANT:MALE -8.677 102.624 -0.085 0.93262

## FED:MALE -417.515 117.665 -3.548 0.00039 ***

## DARK:X4C:MUTANT -203.924 154.367 -1.321 0.18653

## DARK:X4C:FED 359.579 166.436 2.160 0.03076 *

## DARK:MUTANT:FED -34.755 131.950 -0.263 0.79225

## X4C:MUTANT:FED -300.932 155.338 -1.937 0.05274 .

## DARK:X4C:MALE 310.573 179.973 1.726 0.08444 .

## DARK:MUTANT:MALE 174.122 145.194 1.199 0.23047

## X4C:MUTANT:MALE -402.717 159.865 -2.519 0.01178 *

## DARK:FED:MALE 130.230 165.804 0.785 0.43222

## X4C:FED:MALE -59.731 181.594 -0.329 0.74222

## MUTANT:FED:MALE 243.693 146.096 1.668 0.09535 .

## DARK:X4C:MUTANT:FED 538.485 218.393 2.466 0.01370 *

## DARK:X4C:MUTANT:MALE -634.812 225.127 -2.820 0.00482 **

## DARK:X4C:FED:MALE 73.135 255.125 0.287 0.77438

## DARK:MUTANT:FED:MALE -34.333 205.943 -0.167 0.86760

## X4C:MUTANT:FED:MALE 445.454 227.134 1.961 0.04989 *

## DARK:X4C:MUTANT:FED:MALE -236.682 319.106 -0.742 0.45829

**Supplementary Table 7: Energy Expenditure linear regression model summary**

## lm(formula = HEAT.EE ~ DARK * X4C * MUTANT * FED * MALE, data = clams_data)

##

## Residuals:

## Min 1Q Median 3Q Max

## -0.70980 -0.06728 0.00823 0.09020 0.34859

##

## Coefficients:

## Estimate Std. Error t value Pr(>|t|)

## (Intercept) 0.358359 0.007120 50.333 < 2e-16 ***

## DARK 0.041944 0.010061 4.169 3.09e-05 ***

## X4C 0.243589 0.012609 19.319 < 2e-16 ***

## MUTANT 0.002230 0.009987 0.223 0.82335

## FED 0.094419 0.010108 9.341 < 2e-16 ***

## MALE 0.059880 0.012473 4.801 1.61e-06 ***

## DARK:X4C -0.073006 0.017750 -4.113 3.94e-05 ***

## DARK:MUTANT -0.005149 0.014098 -0.365 0.71496

## X4C:MUTANT 0.047228 0.016583 2.848 0.00441 **

## DARK:FED -0.022904 0.014256 -1.607 0.10818

## X4C:FED 0.012185 0.017893 0.681 0.49589

## MUTANT:FED 0.002751 0.014112 0.195 0.84542

## DARK:MALE -0.024107 0.017654 -1.366 0.17212

## X4C:MALE 0.047976 0.019310 2.484 0.01299 *

## MUTANT:MALE -0.018752 0.015505 -1.209 0.22655

## FED:MALE -0.044031 0.017778 -2.477 0.01328 *

## DARK:X4C:MUTANT -0.016739 0.023323 -0.718 0.47296

## DARK:X4C:FED 0.068831 0.025147 2.737 0.00621 **

## DARK:MUTANT:FED 0.004958 0.019936 0.249 0.80360

## X4C:MUTANT:FED -0.042761 0.023470 -1.822 0.06849 .

## DARK:X4C:MALE 0.044737 0.027192 1.645 0.09996 .

## DARK:MUTANT:MALE 0.038421 0.021937 1.751 0.07991 .

## X4C:MUTANT:MALE -0.050369 0.024154 -2.085 0.03707 *

## DARK:FED:MALE 0.027635 0.025051 1.103 0.27001

## X4C:FED:MALE 0.044070 0.027437 1.606 0.10826

## MUTANT:FED:MALE 0.039614 0.022074 1.795 0.07275 .

## DARK:X4C:MUTANT:FED 0.063296 0.032997 1.918 0.05511 .

## DARK:X4C:MUTANT:MALE -0.138255 0.034014 -4.065 4.85e-05 ***

## DARK:X4C:FED:MALE 0.012196 0.038547 0.316 0.75171

## DARK:MUTANT:FED:MALE -0.018846 0.031116 -0.606 0.54476

## X4C:MUTANT:FED:MALE 0.070892 0.034318 2.066 0.03888 *

## DARK:X4C:MUTANT:FED:MALE 0.018946 0.048214 0.393 0.69436

**Supplementary Table 8: Activity linear regression model summary**

Call:

## lm(formula = ACTIVITY ~ DARK * X4C * MUTANT * FED * MALE, data = clams_data)

##

## Residuals:

## Min 1Q Median 3Q Max

## -630.67 -211.61 -78.92 180.72 2282.72

##

## Coefficients:

## Estimate Std. Error t value Pr(>|t|)

## (Intercept) 252.249 18.596 13.565 < 2e-16 ***

## DARK 378.417 26.279 14.400 < 2e-16 ***

## X4C -58.743 32.933 -1.784 0.074511 .

## MUTANT -45.646 26.085 -1.750 0.080175 .

## FED 275.248 26.400 10.426 < 2e-16 ***

## MALE -105.337 32.579 -3.233 0.001228 **

## DARK:X4C -243.513 46.362 -5.252 1.54e-07 ***

## DARK:MUTANT -98.939 36.823 -2.687 0.007227 **

## X4C:MUTANT 73.525 43.315 1.697 0.089645 .

## DARK:FED -347.633 37.236 -9.336 < 2e-16 ***

## X4C:FED -130.379 46.736 -2.790 0.005288 **

## MUTANT:FED -58.302 36.858 -1.582 0.113737

## DARK:MALE -192.026 46.111 -4.164 3.15e-05 ***

## X4C:MALE 53.112 50.436 1.053 0.292346

## MUTANT:MALE 51.335 40.499 1.268 0.204986

## FED:MALE -116.885 46.434 -2.517 0.011847 *

## DARK:X4C:MUTANT -5.054 60.918 -0.083 0.933880

## DARK:X4C:FED 41.960 65.681 0.639 0.522939

## DARK:MUTANT:FED 87.939 52.072 1.689 0.091297 .

## X4C:MUTANT:FED 67.356 61.301 1.099 0.271903

## DARK:X4C:MALE 74.047 71.023 1.043 0.297174

## DARK:MUTANT:MALE 263.622 57.298 4.601 4.27e-06 ***

## X4C:MUTANT:MALE -36.872 63.088 -0.584 0.558931

## DARK:FED:MALE 236.362 65.432 3.612 0.000305 ***

## X4C:FED:MALE 74.972 71.663 1.046 0.295508

## MUTANT:FED:MALE 96.336 57.654 1.671 0.094773 .

## DARK:X4C:MUTANT:FED 88.291 86.185 1.024 0.305660

## DARK:X4C:MUTANT:MALE -221.686 88.843 -2.495 0.012605 *

## DARK:X4C:FED:MALE -9.929 100.681 -0.099 0.921440

## DARK:MUTANT:FED:MALE -126.465 81.272 -1.556 0.119728

## X4C:MUTANT:FED:MALE -119.443 89.635 -1.333 0.182713

## DARK:X4C:MUTANT:FED:MALE -11.886 125.930 -0.094 0.924808

**Supplementary Table 9: Quantification of Phox2b and Islet1 positive cell on the ventral neuroepithlium.**

|  | **% of Phox2b+ cells** | | | **% of positive Islet1+ cells** | | | **% of positive Islet1+ colocalized with Phox2b cells** | | |
| --- | --- | --- | --- | --- | --- | --- | --- | --- | --- |
| **Genotype** | Mean | SD | P-value | Mean | SD | P-value | Mean | SD | P-value |
| Control | 30.7387 | 7.0669 | 0.177 | 10.8290 | 3.1457 | 0.064 | 83.2608 | 9.6527 | 0.554 |
| Nkx2.2^Cre^, Phox2b^Δ8^ | 23.3497 | 8.6550 |  | 5.5191 | 4.5451 |  | 87.8236 | 13.3954 |  |

**References**

1. Hirsch MR, d'Autreaux F, Dymecki SM, Brunet JF, Goridis C (2013) A Phox2b::FLPo transgenic mouse line suitable for intersectional genetics. Genesis.51(7):506-14.

2. Madisen L, Zwingman TA, Sunkin SM, Oh SW, Zariwala HA, Gu H, Ng LL, Palmiter RD, Hawrylycz MJ, Jones AR, Lein ES, Zeng H (2010) A robust and high-throughput Cre reporting and characterization system for the whole mouse brain. Nat Neurosci.13(1):133-40.

3. Matei V, Pauley S, Kaing S, Rowitch D, Beisel KW, Morris K, Feng F, Jones K, Lee J, Fritzsch B (2005) Smaller inner ear sensory epithelia in Neurog 1 null mice are related to earlier hair cell cycle exit. Dev Dyn.234(3):633-50.

4. Nobuta H, Cilio MR, Danhaive O, Tsai HH, Tupal S, Chang SM, Murnen A, Kreitzer F, Bravo V, Czeisler C, Gokozan HN, Gygli P, Bush S, Weese-Mayer DE, Conklin B, Yee SP, Huang EJ, Gray PA, Rowitch D, Otero JJ (2015) Dysregulation of locus coeruleus development in congenital central hypoventilation syndrome. Acta neuropathologica.130(2):171-83.

5. Plummer NW, Evsyukova IY, Robertson SD, de Marchena J, Tucker CJ, Jensen P (2015) Expanding the power of recombinase-based labeling to uncover cellular diversity. Development.142(24):4385-93.

6. Vong L, Ye C, Yang Z, Choi B, Chua S, Jr., Lowell BB (2011) Leptin action on GABAergic neurons prevents obesity and reduces inhibitory tone to POMC neurons. Neuron.71(1):142-54.

7. Vue TY, Bluske K, Alishahi A, Yang LL, Koyano-Nakagawa N, Novitch B, Nakagawa Y (2009) Sonic hedgehog signaling controls thalamic progenitor identity and nuclei specification in mice. J Neurosci.29(14):4484-97.

8. Wang H, Lei Q, Oosterveen T, Ericson J, Matise MP (2011) Tcf/Lef repressors differentially regulate Shh-Gli target gene activation thresholds to generate progenitor patterning in the developing CNS. Development.138(17):3711-21.

9. Zhu H, Aryal DK, Olsen RH, Urban DJ, Swearingen A, Forbes S, Roth BL, Hochgeschwender U (2016) Cre-dependent DREADD (Designer Receptors Exclusively Activated by Designer Drugs) mice. Genesis.54(8):439-46.
