# Supplementary material for: Neonatal apneic phenotype in a murine congenital central hypoventilation syndrome model is induced through non‐cell autonomous developmental mechanisms
Source: Brain Pathol. 2020 Aug 4;31(1):84–102. doi: 10.1111/bpa.12877 (PMC7881415; doi:10.1111/bpa.12877)
Supplement: Supplementary file 13 — Supplementary Material [file BPA-31-84-s010.docx]

**Neonatal Apneic Phenotype in Murine Congenital Central Hypoventilation Syndrome Model is Induced Through Non-Cell Autonomous Developmental Mechanisms**

Correa, Liu, Jones, et al., Supplemental Material

**Supplementary Materials, Methods and Results:**

**Histology**

*Analysis of Olig3 experiments:* Analyses of the nucleus of the solitary tract (NTS) in *Olig3^Cre^, Phox2b^Δ8^* and control littermate P0 pups was challenging due to different atlases using varied terminology. The terms commissural NTS, lateral NTS, and medial NTS are utilized as shown in the Allen Developing Brain Atlas. The term rostral NTS, which is not present in the Allen Brain Atlas, is denoted to represent a population of NTS cells described by Paul Gray that are *LMX1B*-derived (1). Using the Allen Developing Brain Atlas for orientation, sister sections of the hindbrain, cryosectioned at 40 μm, were stained for PHOX2B and Choline Acetyltransferase (ChAT). Dorsal hindbrain structures analyzed, using Neurolucida’s cell counter program, were PHOX2B^+^, ChAT^-^ including the NTS and the area postrema (AP).

*Immunohistochemistry of Embryos:* For experiments investigating rhombomere cytoarchitecture, adult pregnant mice were euthanized with CO_2_ before harvesting mouse embryos by cesarean section. Embryos were drop-fixed in 4% PFA in Diethyl pyrocarbonate treated Phosphate-buffered saline (DEPC-PBS) at 4ºC overnight, equilibrated in 30% sucrose in DEPC-PBS at 4ºC until tissue sank to bottom of container and optimum cutting temperature (OCT) solution (# 23-730-571 Thermo Fisher Scientific)embedded. Identification and orientation of embryonic rhombomere structures were determined using EMAP eMouse Atlas Project (http://www.emouseatlas.org) 3-D serial section reconstruction anatomy models (9). For embryo immunohistochemistry and hybridization, OCT-embedded embryos were transversely cryosectioned at 14µm directly onto a Vectabond-coated slide (#SP-1800, Vector Labs), allowed to dry at room temperature for at least 2 hours, and stored at -80ºC. Embryonic tissue sections were antigen retrieved with 10mM citrate buffer (pH 6.0) in a pressure cooker for 4 minutes then washed with PBS + 0.1% Triton X-100 (#02300221, MP Biomedicals) three times for ten minutes each wash. Tissue was then blocked with 10% horse serum (#06750, Stem Cell Technologies) and 0.1% Triton X-100 in PBS before incubation with primary antibody solution (5% horse serum, 0.1% Triton X-100 in PBS) overnight at 4°C. Tissue was washed with PBS + 0.1% Triton X-100 three times for ten minutes before incubation with secondary antibody solution (5% horse serum, 0.1% Triton X-100) and counterstained with DAPI 1:1000 (#D1306, Invitrogen) for two hours at room temperature in the dark. Sections were first washed with PBS two times then with dH2O once for ten minutes and coverslips were mounted with ProLong Gold Antifade Mountant (#P36934, Invitrogen).

***Rt-PCR Primers:*** Primers were as follows: *Phox2b* splice variant 1 (*Phox2b-201)* FWD, 5’-TCTTCGCTGAGACGCACTAC-3’; *Phox2b-201* REV, 5’-CCTGCTTGCGAAACTTAGCC-3’; *Phox2b* alternative splice variant 2 (*Phox2b-202*) FWD, 5’-ATTGCGTGATTCGTTTGCCC-3’; and *Phox2b-202* REV, 5’-TGGAGTAACAACCCCTTCGTG-3’. *Gapdh* primer sequences were obtained from the RTPrimerDB public database (ID 3429): *Gapdh* FWD, 5’-ACCCAGAAGACTGTGGATGG-3’; *Gapdh* REV, 5’-ACACATTGGGGGTAGGAACA-3’. RT-qPCR was performed on littermate control (*Phox2b^+/+^*) mice.

**Chemogenetic experiments**

*Adult Chemogenetic Physiology Studies:* All data shown in **Figure S4** **– S6**. Animal numbers are included in the figure legends. In the head to head comparisons, we evaluated baseline breathing patterns in the adult chemogenetic experiments, plethysmography data were analyzed by selecting 2-second intervals with the lowest amount of variation in breathing frequency for each 1 minute of recording. These data were then evaluated for comparisons between groups.

*Dimensionality Reduction in Chemogenetic Experiments:* The plethysmography recordings were performed in a fashion blinded by the experimenter. The following plethysmography parameters were utilized: frequency (f), tidal volume (TVb), minute ventilation (MVb), enhanced pause (Penh), pause (PAU), peak inspiratory flow (PIFb), peak expiratory flow (PEFb), inspiratory time (Ti), expiratory time (Te), 50%’ile expiratory flow (EF50), end inspiratory pause (EIP), and end expiratory pause (EEP). The recordings post-CNO were appended to the pre-CNO recording of the same animal, and the resulting dataframe (which included pre and post-CNO measurements) were center scaled. For each of these parameters, we also performed a Poincaré analysis on the scaled data. A Poincaré plot is a scatter graph that visualizes the correlation between two consecutive data points in a time-series (i.e., x-axis: A_n_ versus y-axis: A_n+1_), and is often used to measure respiratory variability (6). We then took the data (center-scaled respiratory data as well as the Poincaré values), and obtained the arithmetic mean and standard deviation, which resulted in 48 features per animal, with two conditions per animal (i.e., the pre-CNO recordings and the post-CNO recordings). Animal genotypes included *Vglut2^cre^*, *ROSA^hM4Di^* (n = 5), *Vglut2^cre^* littermate controls (n = 4), *Nkx2.2^cre^*, *ROSA^hM4Di^* (n= 3), and *Nkx2.2^cre^* littermate controls (n= 3). We then created a 30 X 48 matrix containing each animal’s observation (pre-CNO and post-CNO) as rows and measurements in columns. We then performed a principal component analysis in R using the prcomp function, which successfully reduced the dimensionality, resultin in a 30x30 matrix of principal components. We then calculated the Euclidean Distance between pre-CNO and post-CNO points from the PCA data transformation for each animal by using the dist fuctinon in R. Differences in Euclidean distance for the Nkx2.2 and Vglut2 genoptypes were performed by paired T test, followed by fdr adjustment.

*Newborn respiratory analyses of Nkx2.2^Cre^, Phox2b^Δ8^ and* hM4Di *positive newborn mice:* For the *Nkx2.2^Cre^, Phox2b^Δ8^* animals, the entire recordings were imported into R, and the mean tidal volume (V_T_), minute ventilation (V_E_), respiratory frequency (f_R_), peak respiratory flow (PIF), expiratory flow at 50% expired volume (EF50), inspiratory time (Ti), and expiratory time (Te) was calculated. t-test was performed, and each p-value was then corrected using the p.adjust (method = fdr) function in R.

***Olig3^cre^* Respiratory Physiology, Blood Pressure, and Metabolic Experiments**

*Olig3^cre^ pups:* For analysis of plethysmography recordings on P1 pups, implementations of the Student's t–test occurred by comparing the mean of each respiratory variable during the baseline period and comparing controls to mutants. For the hypercapnic challenges on P21 and P56 mice, biological effects upon each respiratory variable during the transition of baseline to hypercapnia become converted to fold change values by dividing the first quartile of each respiratory variable. For statistical analysis, the mean fold change from control and mutants underwent statistical hypothesis testing by Student's t–test. For the hypoxic challenge on P21 and P56 mice, best-fit curves of frequency (f_R_) and minute ventilation (V_E_) demarcated transition from phase I to phase II of the hypoxic response by calculating the inflexion points. Mean inflexion points from controls and mutants were compared by Student's t–test.

*Chemoreflex analysis following normoxic hypercapnic, hypoxic, and hyperoxic hypercapnic challenges:* P21 and P56 *Olig3^Cre^, Phox2b^Δ8^* animals experienced chemoreflex analysis by plethysmography. Plethysmograph acclimatization occurred by filling the chamber with room air for 30 minutes per day for 3 consecutive days. On the day of recording each mouse underwent acclimatization to the chamber filled with room air for 5 minutes, followed by baseline recordings for 20 minutes. For chemoreflex analysis, hypoxic (8% O2) responses were recorded for 10 minutes, then, after a recovery period of 30 minutes in room air, responses to hypercapnia (7% CO2) were recorded for 10 minutes. In addition, adult mice underwent recordings during hyperoxic hypercapnia. After the 3 days acclimation protocol described above, animals were acclimated again for 30 minutes followed by 15 minutes of baseline recordings in room air. Hyperoxia (65% O2) was induced for 3 minutes prior to an hyperoxic hypercapnia (65% O2 – 8% CO2) challenge recording for 10 minutes. Finally, recovery in room air was recorded for 20 minutes. Recordings were collected during daytime (9 AM and 6 PM) with data points obtained and analyzed every two seconds for the entire recording session.

*Blood pressure measurements:* Blood pressure measurements were performed as described previously (2). The CODA Non-Invasive Blood Pressure System was used for recordings (Kent Scientific Corporation). Mice were individually removed from their cage and held by their tail to allow them to enter the blood pressure tube on their own accord. The O-cuff was then placed as close to the base of their tail as possible or until resistance was felt. The VPR-cuff was placed against the O-cuff. Animals had blood pressure (BP) recordings twice per week for five weeks. The first two weeks were the acclimation phase in which each animal received 30 acclimation cycles, and these recordings were not used for analysis. The second week baseline BPs were recorded for the animals, and each animal received 10 acclimation cycles and 20 regular cycles. On average mice had 14 + 2 accepted cycles.  Usable cycles were then averaged together per mouse. Following two weeks of acclimation the mice had simultaneous baseline recordings performed.  All mice were returned to their home cage for 30 minutes.  Subsequently the mice were then injected with carbachol  for the designed experiment and were immediately placed back in the BP machine for 60 cycles (approximately 35 minutes). Every cycle without motion artifact was used for analysis.  Note for a measurable cycle the animal's volume must be over 15 microliters and there can be no motion artifact from the mouse.

*Metabolic Measurements by CLAMS and statistical modeling:* Whole animal metabolic parameters were measured using the Comprehensive Lab Animal Monitoring System (CLAMS) equipped with a temperature-controlled environmental chamber from Columbus Instruments (Columbus, OH, USA) at room temperature (22 °C) ​​ in cold conditions (4 °C). At each temperature mice were housed and data collected for 24h in the fed state, and 24h fasted. Volume of O_2_ consumption, volume of CO_2_ production, heat production and activity level were measured.  Respiratory-exchange ratio (RER) was calculated as the ratio of total carbon dioxide produced to total oxygen consumed.

To model these effects, a rectangular data table was generated with all animals to evaluate the extent to which the different factors modulated the outcomes. In this approach, VO_2_, VCO_2_, heat energy expenditure, and activity were the response variables, and the predictor factors were the binary outcomes of darkness, cold temperature, mutation status, fed status, or male status. The predictor factors were scored as successes = 1, failures = 0, and were designed as interactors. This technique is powerful as it permits the analysis of factor interaction at the expense of sample size amplification. This resulted in an adjusted R2 of 0.61 for the VO_2_ model, 0.69 for the VCO_2_ model, 0.59 for the heat energy expenditure model, and 0.16 for the activity model (for all models, p < 2X10^-16^). The tables of the interactions are delineated in

*Custom image analysis workflows in R.*

The following custom function was used to identify the efficiency of recombination in *Olig3^cre^*, *Phox2b^Δ8^* animals:

nuclei_count <- function(x, y) {

library(EBImage)

phox2b <- readImage(x)

phox2b <- phox2b[,,1]

phox2b <- na.omit(phox2b)

tomato <- readImage(y)

tomato <- tomato[,,1]

tomato <- na.omit(tomato)

my_auto_thresh <- function(x) {

library(imager)

img <- threshold(x, thr = 'auto')

detach(package:imager)

return(img)

}

tomato_thresh <- my_auto_thresh(tomato)

tomato_brush <- opening(tomato_thresh, makeBrush(5, shape = 'disc'))

tomato_bw <- bwlabel(tomato_brush)

phox2b_thresh <- my_auto_thresh(phox2b)

phox2b_brush <- opening(phox2b_thresh, makeBrush(5, shape = 'disc'))

phox2b_bw <- bwlabel(phox2b_brush)

collage_2 <- computeFeatures.basic(phox2b_bw, tomato_bw, na.rm = TRUE)

df <- as.data.frame(collage_2)

newvar <- (nrow(subset(df, df$b.mean > 0))/nrow(df))*100

merged_image <- rgbImage(green = phox2b_bw, red = tomato_bw)

data <- paste("The percent of PHOX2B positive cells that are also tomato positive equals", as.character(newvar))

writeImage(merged_image, files = paste(x, "merged.jpg"))

mylist = list("data" = data, "phox2b" = phox2b_bw, "tomato" = tomato_bw, 'merged' = merged_image, 'raw tomato' = tomato, 'raw2b' = phox2b)

return(mylist)

}

The following custom function was used to identify the efficiency of recombination in *Olig3^cre^*, *Phox2b^Δ8^* E12.5 animals:

E12.5_analysis<- function( i, p, d, n) {

library(EBImage)

islet = i

p2b = p

dapi = d

nkx = n

kern= (makeBrush(9, shape = "diamond"))

test_i <- thresh(gblur(islet, sigma = 3), 10, 10, 0.012)

test_i <- fillHull(test_i)

test_i <- dilate(erode(test_i, kern))

test_i <-selfComplementaryTopHat(test_i, makeBrush(5, shape= 'Gaussian'))

test_i_bw <- bwlabel(test_i)

segmented_i <- paintObjects(test_i_bw, rgbImage(green = islet*3), col = 'red')

test_2b <- thresh(gblur(p2b, sigma = 3), 10, 10, 0.012)

test_2b <- fillHull(test_2b)

test_2b <-selfComplementaryTopHat(test_2b, makeBrush(5, shape= 'Gaussian'))

test_2b_bw <- bwlabel(test_2b)

segmented_p2b <- paintObjects(test_2b_bw, rgbImage(green = p2b*3), col = 'red')

test_d <- thresh(gblur(dapi, sigma = 3), 10, 10, 0.012)

test_d <- fillHull(test_d)

test_d <-selfComplementaryTopHat(test_d, makeBrush(5, shape= 'Gaussian'))

test_d_bw <- bwlabel(test_d)

segmented_d <- paintObjects(test_d_bw, rgbImage(green = dapi*3), col = 'red')

test_n <- thresh(gblur(nkx, sigma = 5), 10, 10, 0.022)

test_n <- fillHull(test_n)

test_n <-selfComplementaryTopHat(test_n, makeBrush(5, shape= 'Gaussian'))

test_n_bw <- bwlabel(test_n)

segmented_n <- paintObjects(test_n_bw, rgbImage(green = nkx*3), col = 'red')

merged_image = rgbImage(green = test_2b, red = test_i)

df_percent_pos = data.frame("2b positive cells" = corrected_2b_dap, "islet positive cells" = corrected_2islet_dap,

"i+&2b+/2b percent" = percent_islet_pos_phox2b,

'i+&2b+/i' = percent_phox2b_pos_islet)

df_3dplot <- data.frame("mean_islet_2b" = collage_7b[,12], "mean_2b_2b" = collage_6b[,12],

"sd_islet_2b" = collage_7b[,13], "sd_2b_2b" = collage_6b[,13],

"ASM_f_2b" = collage_6b[,25], "ENT_f_2b" = collage_6b[,33])

mylist <- list('data for cell counts' = df_percent_pos, 'data for 3d plot' = df_3dplot,

'islet seg' = segmented_i,

'phox2b seg' = segmented_p2b,

'dapi seg' = segmented_d,

'nkx seg' = segmented_n,

'merged 2b_green_islet_red' = merged_image)

percent_2b_pos_dapi

percent_islet_pos_dapi

percent_islet_pos_phox2b

return(mylist)}

**Justification of Pooling Control Animals with Distinct Genotypes in Nkx2.2^cre^, Phox2b^Δ^8 experiments.**

Figures 1 and 2 of the main text show designation of control animals. We performed a justification for inclusion of controls of different genotypes by analyzing numerical data presented in Figure 2 that was obtained from the head-out plethysmograph instrument. In these experiments, three genotypes were tested: Nkx2.2-, Phox2bΔ8-, Nkx2.2-, Phox2bΔ8+, and Nkx2.2+, Phox2bΔ8+, where controls belong to two classes (Nkx2.2-, Phox2bΔ8-, Nkx2.2-, Phox2bΔ8+), and mutants belong to one class (Nkx2.2+, Phox2bΔ8+). We performed a linear discriminant analysis (LDA) using the MASS package, and plotted the data using ggplot2 (11). In the LDA, we modelled Class (a Factor with levels (i) Nkx2.2-, Phox2bΔ8-, (ii) Nkx2.2-, Phox2bΔ8+, and (iii) Nkx2.2+, Phox2bΔ8+)) as a function of frequency, EF50, inspiratory time, expiratory time, tidal volume, minute ventilation, PEF, and PIF. Note in Supplemental Figure 1 that this results in two axes, showing ~78% separation across LD1 and ~22% separation across LD2. Note that the mutants (blue dots in Supplemental Figure 1) are separated from the two control genotypes across the LD1 axis. The R code for running the LDA and plotting the animals across the LD1 and LD2 axis is shown below:

#importation of data

setwd("/Users/oter04/Documents/Projects/NKX2.2_Project/Manuscript Texts/Resubmission to Brain Pathology 4_21_2020/Figure 2 modifications")

#code for the LDA graph.

library(readxl)

df <- read_excel("nkx2.2_Pup_dataanalysis.xlsx" )

df$Class <- as.factor(df$Class)

library(MASS)

df1 <- df[, 2:11]

scaled_df <- scale(df1)

scaled_df <- as.data.frame(scaled_df)

scaled_df$Class <- df$Class

names(scaled_df)

lda_m <- lda(Class ~ f + EF50+ Ti + Te+TV+MV + PEF + PIF, data = scaled_df)

new_data <- predict(lda_m, scaled_df)

library(ggplot2)

class(new_data$x)

lda_data <- data.frame(new_data$x)

lda_data$Class <- df$Class

lda_data

p <- ggplot(data = lda_data, aes(x = LD1, y = LD2)) +

geom_point(size = 3, aes(col = factor(Class))) +

xlab("LD1 (76.29%)") +

ylab("LD2 (23.71%") +

ggtitle("Linear Discriminant Analysis")+

theme_bw()+

theme(plot.title = element_text(face = "bold"),

axis.title.x = element_text(face = "bold"),

axis.title.y = element_text(face = "bold"),

axis.text.x = element_text(face = "bold"),

axis.text.y = element_text(face = "bold"))

We next sought to determine the cluster designation using a machine learning approach. In our approach, we removed the class label "NKX2.2-P2bD8-", which resulted in a dataset containing only one control class ("NKX2.2-P2bD8+") and one mutant class ("NKX2.2+P2bD8+"). We utilized a random forest with 1000 trees from the randomForest package (4), support vector machines with a radial kernel from the e1071 package, an LDA from the MASS package, and a k-nearest neighbor with k = 3 from the class package. We then passed the data from the subsetted "NKX2.2-P2bD8-" data, and evaluated the output from the various models. All of the models predicted that the data from the "NKX2.2-P2bD8-" dataset was control. These data are shown in Supplemental Figure 1B. We conclude that data from both control genotypes can be pooled for our analyses. The R code we used for these designations follows:

class2 <- subset(df, df$Class != "NKX2.2-P2bD8-")

class1 <- subset(df, df$Class == "NKX2.2-P2bD8-")

class2 <- droplevels(class2)

class1 <- droplevels(class1)

levels(class2$Class)

set.seed(1123)

library(randomForest)

rf_model <- randomForest(Class ~ ., data = class2, ntree = 1000)

output <- predict(rf_model, class1, type = "prob")

output

library(e1071)

svm_model <- svm(Class ~ ., data = class2, kernel = "radial")

output_svm <- predict(svm_model, class1, type = "prob")

output_svm

lda_model2 <- lda(Class ~ ., data = class2)

output_lda <- predict(lda_model2, class1)

output_lda$class

library(class)

train = class2[,2:11]

test= class1[,2:11]

knn_model <- knn(train,test, k = 3, cl = class2$Class, prob = TRUE)

knn_model

**Animal Treatments, Automated Image Analysis, and Machine Learning Workflow for Cell Cycle Experiments.**

For image analysis, CldU (2.5 hour total pulse) and EdU (0.5 hour total pulse) treated animals from littermate control and *Nkx2.2^cre^, Phox2b^Δ8^* animals were cryosectioned and immunostained with anti-BrdU antibody, anti-Phox2b antibody, and also exposed to the Click-IT reaction to detect EdU. To visualize nuclei, sections were stained with DAPI. Images were captured at 20x Z-stack confocal images and saved as .czi files. Image capturing was performed blinded to genotype. The .czi files were imported into FIJI. For the BrdU and EdU analysis over dapi, the pMNv domain was demarcated manually and rhombomere 4 was defined as a region where the ventral neuroepithelium showed Phox2b expression from the ventricular epithelium to the pial surface (note that r2/3 and r5 do not show Phox2b expression at the ventricular surface). Using the manual cell counter feature in FIJI, we quantified blindly the proportion of BrdU positive cells (over DAPI), quantified the proportion of BrdU+EdU- cells (over DAPI), and then quantified the proportion of EdU+ cells (over DAPI). We then calculated S-phase length using the DA method as implemented by Ponti, et al (8). For ease of illustration, all values were pooled and scaled in R using the “scale” function set to default parameters. The data was illustrated as a density plot of scaled units (x-axis) and kernel density function (y-axis) using the “density” function in R set to default parameters. Control versus mutant values were tested by t-test and the corresponding p-values were illustrated on the top right of the figure legend (**Figure 7**). To quantify the proliferative populations in pMNv Phox2b+ we utilized an unbiased, automated approach using the EBImage package from R (7) using the following steps. **Step 1:** Z-stacked 20x confocal images of the neuroepithelium stained with Dapi/Phox2b/EdU/BrdU were opened in FIJI, underwent average intensity projection of the Z-stack, split into channels and saved as .tif images. **Step 2:** The .tif images were then imported into Rstudio. First the image was converted to Grayscale, and then a medianFilter function was passed with a kernel size = 2. Next, adaptive thresholding was applied with width and height = 5, offset = 0.02. Then the morphological operation selfComplementaryTopHat was performed with a makeBrush set to size = 5, shape = Gaussian, followed by a fillHull operation. The nuclear mask was created by passing the image through the watershed command on the distance map (distmap) of the processed image with tolerance = 1, and ext = 1. These parameters were developed empirically on known control samples. The nuclear mask of the phox2b stained images was then overlayed onto grayscaled .tif images of the original phox2b, BrdU, and EdU stained images and the computeFeatures function was called. The dataframes where then combined with features from Phox2b data, BrdU data, and Edu data. We size excluded the objects by only selecting objects with an area greater than 10**. Step 3:** All objects were graphed on a cartesean plane and for each image the dataframe was passed through a kmeans algorithm to obtain only objects within the pMNv domain. Some objects were then excluded manually by location. **Step 4:** Having selected pMNv objects, the dataframe objects were labelled as either belonging to Experimental Class (i.e., *Nkx2.2^cre^, Phox2b^Δ8^* animals) or Control Class (littermate negative controls). This large dataset contained over 20,000 objects from both classes. We then selected the following features, which based on prior experience are often capable of separating objects. These features included: object area, object eccentricity, object perimeter, Phox2b channel pixel intensity standard deviation, Phox2b channel mean pixel intensity ,the lowest Phox2b channel 5^th^ percentile pixel intensity, Phox2b channel entropy, Phox2b channel variance, EdU channel mean pixel intensity, EdU channel 5^th^ percentile pixel intensity, EdU channel pixel intensity standard deviation, EdU channel entropy, EdU channel variance, BrdU channel mean pixel intensity, BrdU channel pixel intensity standard deviation, BrdU channel 5^th^ percentile pixel intensity, BrdU channel variance, BrdU channel SAV. These condensed features were then utilized to test the hypothesis that the phox2b segmented objects were different between groups. To this end, we randomly sampled into 70% for training, and 30% for testing. We then tested the following classifier algorithms. RandomForest (5), adaboost from the Joust package, support vector machines from the e107 package, and LDA from the MASS package. Hyperparameters for the SVM were kernel = polynomial, randomForest had 1000 trees, and the adaboost had a tree depth of 2 and 500 rounds of boosting. After training these algorithms, their capability of correctly classifying data was determined by passing the test data through the predictive models. A confusion matrix was then tested using the confusionMatrix function of the caret package (3). The outcome of the confusion matrix is presented graphically in Figure S10. In each experiment, the no information rate was 57%, and the positive class was Control. Note that the random forest algorithm had the greatest accuracy at up to 74%, with the support vector machine have the greatest specificity rated at 94%. AUC of an ROC analysis was 0.73 (calculated by the roc function in the pROC package). Despite the relatively low accuracy, the probability that the accuracy was greater than the no information rate in the random forest and adaboost models was p < 10^-16^. This exploratory step indicating that there were differences between control and mutant Phox2b segmented objects. Upon evaluation the feature importance, we found that BrdU mean intensity had the largest mean decrease in gini (**Figure S10E**).

**Supplementary Figure Legends**

**Figure S1: Justification for pooling of control genotypes.**

(A) Linear discriminant analysis of genotype analysis utilizing data presented in Figure 2 of the main manuscript. Note that the genotypes are color coded and are composed of two control classes (red and green dots) and one mutant class (blue dots). 76.29% of the data is separated across the LD1 axis, with 23% of the data separated across the LD2 axis. Note that the blue dots cluster on right of graph, and green and red dots cluster on left of graph. (B) Results of the machine learning predictions from algorithms trained on data from one control genotype class and one mutant class. Remaining data (n = 2) from the second control genotype were passed into the RF (random forest), SVM (support vector machine, radial kernel), LDA, and knn. All models predicted that the control genotype tested belonged to the control group.

**Figure S2. Respiratory Physiology of *Olig3^Cre^, Phox2b^Δ8^* Mice**

Control mice data are in gray, *Olig3^Cre^, Phox2b^Δ8^* mutant mice data are in red.  Ages of the experimental groups are denoted in each sub-heading, with (A-F) representing baseline respiratory physiology performed in restrained/head out P1 pups for control (n=9) or *Olig3^Cre^, Phox2b^Δ8^*  (mutant n=11) mice, (G-L) representing P21 recordings (control n= 8; mutant n=12), and (M-R) representing P56 recordings (control n=9; mutant n=12). In the P1 experiments, measurements include the mean of baseline room air recordings for frequency (f_R_) (A), tidal volume (V_T_) (B), minute ventilation(V_E_) (C), inspiratory time (Ti)(D), expiratory time (Te)(E), and expiratory flow at 50% of expiration (EF50) (F). In g-r, all mouse data were pooled for the two groups and analyzed to generate smoothed best fit curves plots of baseline versus hypercapnic challenge, or to plot the entire hypoxic challenge delineating frequency (f_R_), tidal volume (V_T_) and minute ventilation(V_E_).  Each graphic contains a title delineating the experimental measurements.  The hypoxia challenges show the characteristic roll-off phase of CNS-mediated respiratory repression that settles to a baseline. These best-fit curves were generated in Rstudio by linear regression, and we calculated the inflection point by determining the value at which dy/dt =0.  No statistical differences were seen between controls and mutants with regards to fold increase during hypercapnic challenge or in the baseline value during hypoxia challenge. Box plots show solid black line to represent the median, the box represents in the interquartile range, and the whiskers represent 1.5 times the interquartile range.

**Figure S3. Hyperoxic hypercapnic challenge *in Olig^Cre^, Phox2b^Δ8^* mice.**

Adult mice were subjected to whole-body plethysmography to evaluate respiratory physiology under an hyperoxic hypercapnic challenge. Control mice data are in gray (n=3), *Olig3^Cre^, Phox2b^Δ8^* mutant mice data are in red (n = 4). In order to compare baseline versus hyperoxic hypercapnic challenge, data from all of the mice analyzed were pooled for the two groups analyzed to generate smoothed best fit curves plots of frequency (A), tidal volume (B), minute ventilation (C), inspiratory time (D), expiratory time (E), peak inspiratory flow (F), peak expiratory flow (G), (Rpef) (H), and EF50 (I). No statistical differences were seen between controls and mutants regarding fold increase during hyperoxic hypercapnic challenge. Box plots show solid black line to represent the median, the box represents in the interquartile range, and the whiskers represent 1.5 times the interquartile range.  (f_R_ = frequency, V_T_ = tidal volume, V_E_ = minute ventilation, Ti = inspirator time, Te = expiratory time, PIFb = peak inspiratory flow, PEFb = peak expiratory flow, Rpef = fraction on expiratory time where the peak occurs, EF50 = expiratory flow at 50% of expiration.

**Figure S4. F, TV, and MV Analysis Following Chemogenetic Silencing of *Nkx2.2*-derived cells.**

Dot plots show genotype on top, parameter on Y-axis (either frequency, tidal volume, or minute ventilation), and time on X-axis for sample animals before and after i.p. injection of CNO (0.25 mg/kg). *Nkx2.2^Cre^*, *ROSA^+/h4MDI^* animals are plotted in blue for dot plots and bar graphs, and *Vglut2^Cre^*, *ROSA^+/h4MDI^* animals are plotted in purple dot plots and bar graphs. Arithmetic means of the parameters pooled from all animals are shown in c1, i1, o1 for *Nkx2.2^Cre^*, with c2, i2, and o2 showing the standard deviation of these animals. Arithmetic means of the parameters pooled from all animals are shown in F1, L1, Q1 for *Vglut2^Cre^*, with F2, L2, and Q2 showing the standard deviation of these animals. p-values of no significance are shown in the box plots in black font, with corrected p-values of < 0.05 shown in red. Box plots show solid black line to represent the median, the box represents in the interquartile range, and the whiskers represent 1.5 times the interquartile range. In each dot plot, the experimental paradigm is shown delineating the CNO treatment. Note that data were appended from pre and post-CNO recordings and plotted continuously on the same graph for ease of representation. For *Nkx2.2* experiments, *Nkx2.2^Cre^, ROSA^+/h4MDI^* n = 3, control animals n = 3. For *Vglut2* experiments, *Vglut2^Cre^, ROSA^+/h4MDI^* n = 4, control n = 5. Adjusted p-values were obtained by using the p.adjust function call in Rstudio.

**Figure S5. Ti, Te, PIF, PEF, and EF50 Analysis Following Chemogenetic Silencing of *Nkx2.2*-derived Cells**

Dot plots show genotype on top, parameter on Y-axis (eg., inspiratory time, expiratory time, peak inspiratory flow, peak expiratory flow, and EF50), and time on X-axis for sample animals before and after i.p. injection of CNO. *Nkx2.2^Cre^*, *ROSA^h4MDI^* animals are plotted in blue for dot plots and bar graphs, and *Vglut2^Cre^*, *ROSA^h4MDI^* animals are plotted in purple dot plots and bar graphs. Arithmetic means of the parameters pooled from all animals are shown in C1, I1, O1, U1, aa1 for *Nkx2.2^Cre^*, with C2, I2, O2, U2, AA2 showing the standard deviation of these animals. Arithmetic means of the parameters pooled from all animals are shown in F1, L1, R1, X1, DD1 for *Vglut2^Cre^*, with F2, L2, R2, X2, and DD2 showing the standard deviation of these animals. P-values of no significance are shown in the box plots in black font, with corrected *p*-values of < 0.05 shown in red. Box plots show solid black line to represent the median, the box represents in the interquartile range, and the whiskers represent 1.5 times the interquartile range. In each dot plot, the experimental paradigm is shown delineating the CNO treatment. Note that data were appended from pre and post-CNO recordings and plotted continuously on the same graph for ease or representation. For *Nkx2.2* experiments, *Nkx2.2^Cre^*, *ROSA^h4MDI^* n = 3, control animals n = 3. For Vglut2 experiments, *Vglut2^Cre^, ROSA^h4MDI^* n = 4, control n = 5.

**Figure S6. PAU, PENH, EEP, EIP Analysis Following Chemogenetic Silencing of *Nkx2.2*-derived Cells**

Dot plots show genotype on top, parameter on Y-axis (eg., PAU, PENH, EEP, and EIP), and time on X-axis for sample animals before and after i.p. injection of CNO. *Nkx2.2^Cre^*, *ROSA^h4MDI^* animals are plotted in blue for dot plots and bar graphs, and *Vglut2^Cre^, ROSA^h4MDI^* animals are plotted in purple dot plots and bar graphs. Arithmetic means of the parameters pooled from all animals are shown in C1, I1, O1, and U1, for *Nkx2.2^Cre^*, with C2, I2, O2, and U2 showing the standard deviation of these animals. Arithmetic means of the parameters pooled from all animals are shown in F1, L1, R1, and X1 for *Vglut2^Cre^*, with F2, L2, R2, and X2 showing the standard deviation of these animals. P-values of no significance are shown in the box plots in black font, with corrected p-values of < 0.05 shown in red. Box plots show solid black line to represent the median, the box represents in the interquartile range, and the whiskers represent 1.5 times the interquartile range. In each dot plot, the experimental paradigm is shown delineating the CNO treatment. Note that data were appended from pre and post-CNO recordings and plotted continuously on the same graph for ease or representation For *Nkx2.2* experiments, *Nkx2.2^Cre^*, *ROSA^+/h4MDi^* n = 3, control animals n = 3. For Vglut2 experiments, *Vglut2^Cre^, ROSA^+/hM4DI^* n = 4, control n = 5.

**Figure S7. Respiratory Function Analysis Following Chemogenetic Silencing of *Nkx2.2*-derived circuits in newborn mice.**

Newborn pups were subjected to restrained (head out) plethysmography and frequency, tidal volume, minute ventilation, Ti, and EF50 were plotted. Box whisker plots in A1, B1, C1, D1, and E1 graph the fold change in means for each animal (pre-CNO mean divided by post-CNO mean) in control (gray) and *Nkx2.2^Cre^*, *ROSA^h4MDI^* (blue) groups. Box plots show solid black line to represent the median, the box represents in the interquartile range, and the whiskers represent 1.5 times the interquartile range. A2, B2, C2, D2, and E2 show the fold change in standard deviations of the groups (i.e., pre-CNO 1^st^ quartile divided by post-CNS 1^st^ quartile, or pre-CNO s.d. divided by post-CNO s.d.). Each parameter tested in each graph is delineated by the title. Genotypes are delineated in the X-axis. *Nkx2.2^Cre^*, *ROSA^h4MDI^* n = 3, control animals n = 11.

**Figure S8. Neuropathological Findings in *Olig3^Cre^, Phox2b^Δ8^* mice.**

Panels A-C and I demonstrate the neuropathology of *Olig3^Cre^, Phox2b^Δ8^* mice. In A1-A3, the nVII is shown, in b1-b3, the DMNV, and in C1-C3 the area postrema. Box-whisker plots in A3, B3, and C3 show median with black bar, and interquartile ranges at the extremes of the box. Black dots represent the actual values of each animal (a-c is performed in animals at age P0/P1, n = 3 control, n = 3 *Olig3^Cre^, Phox2b ^Δ8^*). Evaluation of the Olig3 lineage was performed by interbreeding *Olig3^Cre^* mice with *ROSA^tdTomato^* mice (panels d- h2 and panel j). Panels d-g are generated from P0/P1 mice, panels H1-H2 from P7 mice. The NA and nVII are not *Olig3*-derived. However, the area postrema and dorsal ependymal layer show strong *Olig3* derivation. Only a subset of PHOX2B-positive neurons in the NTS are *Olig3* derived. Panel i shows the quantification of PHOX2B-positive cells in the caudal NTS to rostral NTS. X axis = 0 denotes obex, with the negative values indicating a rostral direction in micrometers, and the + direction indicating caudal in micrometers. More PHOX2B positive cells are present in the *Olig3^Cre^, Phox2b^Δ8^* mice relative to controls in slices 240 μm and 160 μm rostral to obex (* p < 0.05, n = 3 control, n = 3 *Olig3^cre^, Phox2b^Δ8^*). Panel J shows quantification per section of PHOX2B+ and tdTomato+ cells/tdTomato + cells. The P0/1 animals are in red dots, p7 animals are in black dots. The solid red and black lines are the best fit curves obtained from a linear regression estimation. In P0/1 animals, efficiency is significantly higher in the rostral NTS, peaking at an estimated 15% efficiency in PHOX2B-positive cells in the rostral NTS. IS = inferior salivary nucleus (10) rNTS = rostral extension of NTS as defined by Gray (1); mNTS = NTS, medial part, lat-NTS = NTS, lateral part; cNTS = commissural NTS. mNTS, lat-NTS, and cNTS were defined using Allen Brain Atlas.

**Figure S9. Cardiovascular Physiology of *Olig3^Cre^, Phox2b^Δ8^* Mice.**

Control mice data are in gray, *Olig3^Cre^, Phox2b^Δ8^* mutant mice data are in red. Blood pressure from adult mice (24 weeks old) from each genotype (control n=3; mutant n= 5) were recorded during baseline conditions and after carbachol injection. Mean systolic pressure (a); mean diastolic pressure(b) and mean blood pressure (c) for each genotype group before and after carbachol injection. Two-way repeated measures ANOVA was used for statistical analysis showing significant differences when comparing blood pressure before and after carbachol injection, but no significant differences were observed between genotypes.

**Figure S10: Machine Learning analysis of Cell Cycle Data.**

Results of the confusion matrix are plotted for (A) random forest , (B) Adaboost, (C) support vector machine, and (D) linear discriminate analysis. Results of the confusion matrix plotted using the fourfoldplot function in R, which plots a 2 by 2 table shown as a quarter circle, so that the area of the semicircle in the quadrant is proportional to the frequency. Confidence intervals are highlighted by the rings within the semicircle. NIR = no information rate. CI = confidence interval. (E) Barplot of the mean decrease gini coefficients obtained from the random forest classifier. Note that BrdU mean intensity, and standard deviation of BrdU mean intensity represent the most important features in successful classification.

**Figure S11: GFP expression analysis in *Nkx2.2^cre^* and *Olig3^cre^* drivers.**

Evaluation of GFP expression in *Cre-*positive, *PHOX2B^Δ8^* mice are shown control mice (A1, B1, and C1), *Nkx2.2^Cre^, Phox2b^Δ8^* E10.5 embryos (A2, B2, C2), and in the dorsal medulla of *Olig3^Cre^, Phox2b^Δ8^* at P0/1. GFP is noted in animals but not *Nkx2.2^Cre^, Phox2b^Δ8^* animals.

References

1. Gray PA (2013) Transcription factors define the neuroanatomical organization of the medullary reticular formation. Front Neuroanat.7:7.

2. Kempton A, Cefalu M, Justice C, Baich T, Derbala M, Canan B, Janssen PML, Mohler PJ, Smith SA (2018) Altered regulation of cardiac ankyrin repeat protein in heart failure. Heliyon.4(1):e00514.

3. Kuhn M (2019) caret: Classification and Regression Training.

4. Kursa MB, Rudnicki WR (2010) Feature Selection with the Boruta Package. J Stat Softw.36(11):1-13.

5. Liaw A, Wiener M (2002) Classification and Regression by randomForest. R News.2002(2):18-22.

6. Mellen NM, Janczewski WA, Bocchiaro CM, Feldman JL (2003) Opioid-induced quantal slowing reveals dual networks for respiratory rhythm generation. Neuron.37(5):821-6.

7. Pau G, Fuchs F, Sklyar O, Boutros M, Huber W (2010) EBImage--an R package for image processing with applications to cellular phenotypes. Bioinformatics.26(7):979-81.

8. Ponti G, Obernier K, Guinto C, Jose L, Bonfanti L, Alvarez-Buylla A (2013) Cell cycle and lineage progression of neural progenitors in the ventricular-subventricular zones of adult mice. Proc Natl Acad Sci U S A.110(11):E1045-54.

9. Richardson L, Venkataraman S, Stevenson P, Yang Y, Moss J, Graham L, Burton N, Hill B, Rao J, Baldock RA, Armit C (2014) EMAGE mouse embryo spatial gene expression database: 2014 update. Nucleic Acids Res.42(Database issue):D835-44.

10. Stornetta RL, Moreira TS, Takakura AC, Kang BJ, Chang DA, West GH, Brunet JF, Mulkey DK, Bayliss DA, Guyenet PG (2006) Expression of Phox2b by brainstem neurons involved in chemosensory integration in the adult rat. J Neurosci.26(40):10305-14.

11. Wickham H (2016) ggplot2: Elegant Graphics for Data Analysis, Springer: New York, New York.
