# Supplementary material for: Neonatal apneic phenotype in a murine congenital central hypoventilation syndrome model is induced through non‐cell autonomous developmental mechanisms
Source: Brain Pathol. 2020 Aug 4;31(1):84–102. doi: 10.1111/bpa.12877 (PMC7881415; doi:10.1111/bpa.12877)

Alzate, Liu et al., Supplemental FIgure 6

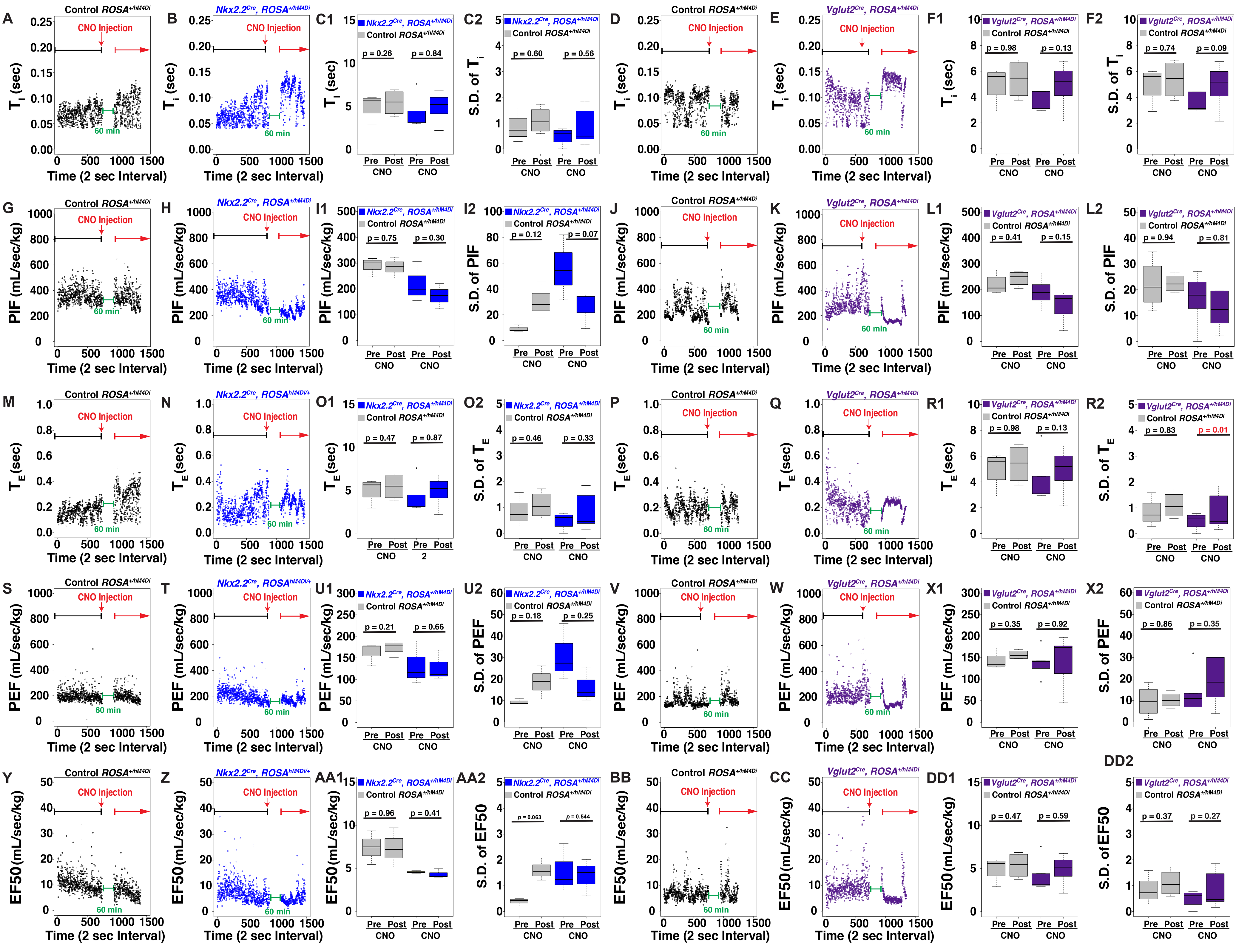

Supplement: Supplementary file 6 — Figure S6. PAU, PENH, EEP, EIP analysis following chemogenetic silencing of Nkx2.2‐derived cells. [file BPA-31-84-s003.pdf]
